# Direct C(sp^3^)–H allylation of 2-alkylpyridines with Morita–Baylis–Hillman carbonates via a tandem nucleophilic substitution/aza-Cope rearrangement

**DOI:** 10.3762/bjoc.17.167

**Published:** 2021-10-01

**Authors:** Siyu Wang, Lianyou Zheng, Shutao Wang, Shulin Ning, Zhuoqi Zhang, Jinbao Xiang

**Affiliations:** 1 The Center for Combinatorial Chemistry and Drug Discovery, School of Pharmaceutical Sciences, Jilin University, 1266 Fujin Road, Changchun, Jilin 130021, P. R. China

**Keywords:** 2-alkylpyridines, allylic alkylation, aza-Cope rearrangement, catalyst-free, Morita–Baylis–Hillman carbonates

## Abstract

A base- and catalyst-free C(sp^3^)–H allylic alkylation of 2-alkylpyridines with Morita–Baylis–Hillman (MBH) carbonates is described. A plausible mechanism of the reaction might involve a tandem S_N_2’ type nucleophilic substitution followed by an aza-Cope rearrangement. Various alkyl substituents on 2-alkylpyridines were tolerated in the reaction to give the allylation products in 26–91% yields. The developed method provides a straightforward and operational simple strategy for the allylic functionalization of 2-alkypyridine derivatives.

## Introduction

**Scheme 1:**
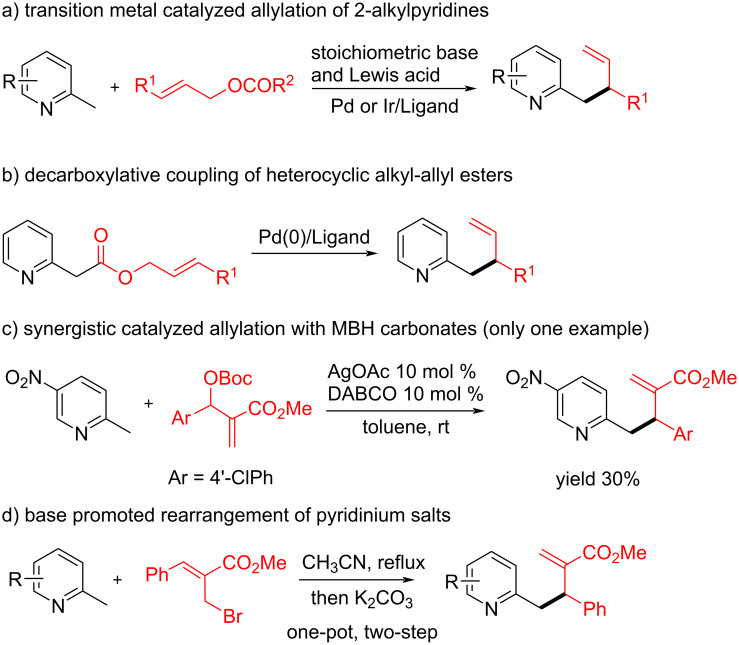
The benzylic C(sp^3^)–H allylic alkylation reactions of 2-alkylpyridines.

Pyridines are among the most important heterocyclic structural moieties in many biologically active natural products, pharmaceuticals, and agrochemicals [1–3]. Therefore, the development of efficient strategies for functionalized pyridine derivatives would be significant in many fields [4–7]. Although transition-mental-catalyzed allylic substitution reactions employing various nucleophilic or electrophilic allylic precursors have been extensively studied [8–10], limited strategies were reported for their application in the C(sp^3^)–H allylic functionalization of 2-alkylazaarenes. Due to the high p*K*_a_ value of alkyl azaarenes, the functionalization of benzylic C(sp^3^)–H was challengeable and pre-activation of the benzylic proton with suitable Lewis acids was often required prior to deprotonation of the alkyl chain by a stoichiometric base (Scheme 1a) [11–17]. In most cases, super stoichiometric amounts of strong bases, such as *n*-BuLi or LiHMDS, would be used, which severely limited their applications. Another strategy requires a pre-existing electron-withdrawing carboxylate groups to further activate the pyridylic C(sp^3^)–H bond (Scheme 1b). For examples, Tunge et al. developed a Pd-catalyzed intramolecular decarboxylative coupling of heterocyclic ally esters via a tandem allylation/Cope rearrangement strategy [18]; Hartwig and co-workers reported a stereo-divergent allylic substitution with azaarene acetamides and acetates catalyzed synergistically by a metal acyclic iridium complex and a chiral Cu(I) complex [19].

Besides transition-metal-catalyzed allylic substitution reactions, Lewis-base-catalyzed allylic functionalizations using Morita−Baylis−Hillman (MBH) adducts as electrophilic allylic precursors have also been widely explored in the past decades. These reactions typically proceed under the catalysis of a nucleophilic tertiary amine or phosphine, and no metal catalysts are needed, which makes this strategy more synthetically useful for the construction of allyl-substituted scaffolds. However, these reactions were mainly suitable to strong nucleophiles such as malonates, amines, or alcohols etc. [20–23]. The allylic substitution reactions using some weak nucleophiles, such as alkyl aza-arenes, were very limited [24–29]. In 2014, Rios and coworkers developed a synergistic catalyzed allylic alkylation between electron-deficient 2-ethyl benzoxazoles and MBH carbonates by the combination of a Lewis base and a metal salt [24]. In their studies, although pyridine derivatives were also applicable in the reaction, the presence of a strong electron-withdrawing NO_2_ group was necessary to promote the reaction but only with low yield (30%) (Scheme 1c). Recently, electron-deficient 3,5-dimethyl-4-nitroisoxazole and 2-methyl-3-nitroindoles were also served as vinylogous pronucleophiles to proceed the asymmetric allylic alkylation with MBH carbonates [25–26]. Overall, the reported C(sp^3^)–H allylic substitution reactions were mainly applied on the 2-alkyl-azaarenes containing strong electron-withdrawing groups. While the reactions with inactive alkyl-aza-arenes were rare [30–31]. Kim et al. have reported a K_2_CO_3_-promoted one-pot allylation reaction of 2-alkylpyridines with MBH-derived allyl bromides (Scheme 1d) [30]. This reaction was assumed to involve the deprotonation of the initially formed 2-methylpyridinium salt by base to generate an *N*-allyl enamine intermediate, which undergoes a 3-aza-Cope rearrangement to give the allyl-substituted products. To the best of our knowledge, there were no reports on the direct C(sp^3^)–H allylic alkylation of inactive 2-alkylpyridines employing MBH carbonates as the allylic precursors.

**Scheme 2:**
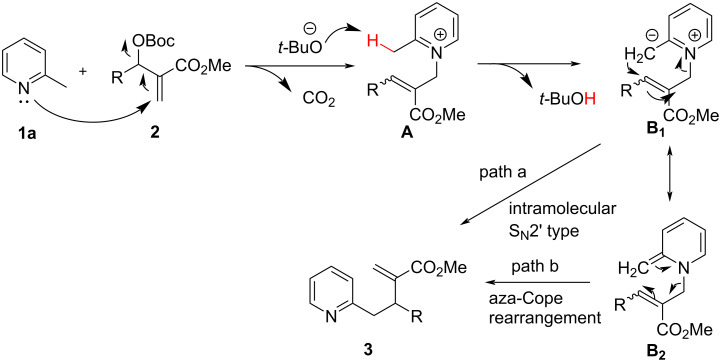
Mechanistic hypothesis of the alkylation reaction of 2-alkylpyridines with MBH carbonates.

Inspired by the previous work from Kim’s group [30] and based on our continual interest in the application of MBH adducts [32–33], herein we present our studies on a direct benzylic allylic reaction of 2-alkylpyridines with MBH carbonates as allylic precursors under base- and catalyst-free conditions. As depicted in Scheme 2, we envisioned that the MBH carbonate **2** can undergo the S_N_2’ reaction with the nucleophilic 2-picoline **1a**, giving the pyridinium cation intermediate **A.** The in situ generated *tert*-butoxide anion has enough basicity to deprotonate of the activated benzylic proton of intermediate **A** and generates the anion **B_1_**, which can be tautomerized into more stable enamine intermediate **B_2_**. Finally, the intermediates **B_1_** or **B_2_** occur an intramolecular S_N_2’ type reaction (path a) or an aza-Cope rearrangement under thermal conditions to generate product **3**.

## Results and Discussion

To evaluate our idea for the allylic alkylation reaction of MBH carbonates to 2-alkylpyridines, 2-picoline (**1a**) and MBH carbonate **2a** were selected as the model substrates to optimize the reaction conditions. Considering no additional reagents were needed in the reaction, only the solvents were screened initially. In fact, the reaction was performed just by heating the mixture of substrates **1a** and **2a** (**1a**/**2a** = 2:1) in solvents at the indicated temperature. Among the solvents tested, CH_3_CN was found to be the best reaction medium. The reaction proceeded smoothly in CH_3_CN at 80 °C and completed within 4 hours to give the desired allylation product **3a** in 91% isolated yield (Table 1, entry 1). While weak polar solvents such as toluene, 1,2-DCE or dioxane led to longer reaction time and gave lower yield although the reactions were carried out at higher or similar temperature (Table 1, entries 2–4). Aprotic DMF was also a suitable solvent, and the reactions could be completed within about 2 hours but with slightly lower yields (Table 1, entry 5 and 6). Thus, CH_3_CN was selected as the optimum solvent. Using CH_3_CN as the solvent, we also attempted the reaction at room temperature, but failed to give any products (Table 1, entry 7). It was also found that an excess amount of 2-picoline (**1a**, 2 equiv) was necessary to ensure the full conversion of **2a**. Decreasing the amount of **1a** would lead to incomplete reaction or longer reaction time (the results were not listed). Hence, the optimal conditions were chosen by performing the reaction in CH_3_CN at 80 °C with the molar ratio 2:1 of **1a** and **2a**.

**Table 1:** Optimization of the reaction conditions for the allylic alkylation of **1a**.^a^

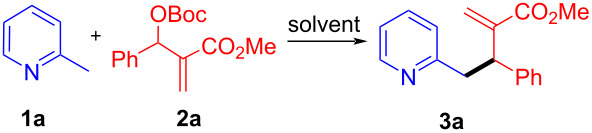				

Entry	Solvent	Temp. (°C)	Time (h)	Yield (%)^b^

1	CH_3_CN	80	4	91
2	toluene	110	24	85
3	1,2-DCE	80	24	49
4	dioxane	100	10	78
5	DMF	100	2	70
6	DMF	80	2.5	67
7	CH_3_CN	rt	24	N.R.

^a^All reactions were carried out with **1a** (1.0 mmol, 2 equiv) and **2a** (0.5 mmol, 1 equiv) in the given solvent (2.0 mL) at the indicated temperature at N_2_ atmosphere; ^b^isolated yield.

**Scheme 3:**
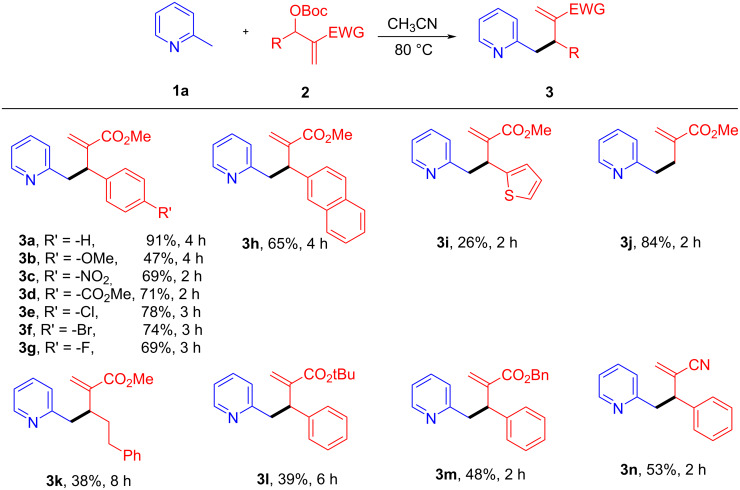
Scope of MBH carbonates **2** with 2-picoline **1a**. The reactions were performed using **1a** (1.0 mmol, 2 equiv) and MBH carbonate **2** (0.5 mmol, 1 equiv) in CH_3_CN (2 mL) at 80 °C. Yields are determined after silica gel column chromatography. EWG = electron withdrawing group.

With the optimal conditions in hand, the substrate scope and limitation of the allylic alkylation were studied. As depicted in Scheme 3, a wide range of MBH carbonates **2** could proceed the allylation reaction with 2-picoline (**1a**). The MBH carbonates **2** containing electron-withdrawing groups on the phenyl ring, such as -NO_2_, -CO_2_Me, and halogen etc., were examined and were found to be well tolerated, providing the desired products in moderate to good yields (**3c**–**g**, yield 69–78%). Compared with electron-withdrawing groups, the presence of electron-donating groups led to a slightly sluggish reaction and a low reaction yield was observed for 4-MeO substituted carbonate (**3b**, yield 47%). The MBH carbonate containing more steric demanding 2-naphthyl group also did not significantly influence the reaction, providing the corresponding product **3h** in 65% yield. However, the MBH carbonate with a 2-thienyl group only gave only a low yield of the product **3i** (yield 26%). Besides aromatic substituents, the reactions of the MBH carbonates with H or alkyl groups were also investigated. For the carbonate with R = H, the reaction worked pretty well to give the mono-allylic product **3j** in 84% yield. However, when R = Ph(CH_2_)_2_- as an example, a longer reaction time was needed and the product **3k** was obtained in lower yield (38%). Finally, the possibility to modify the electron-withdrawing groups (EWG) in **2** was also checked. It was found that the reactions still worked in all of the cases although lower reaction yields were obtained (**3l**, **3m** and **3n**, 39–53% yields).

**Scheme 4:**
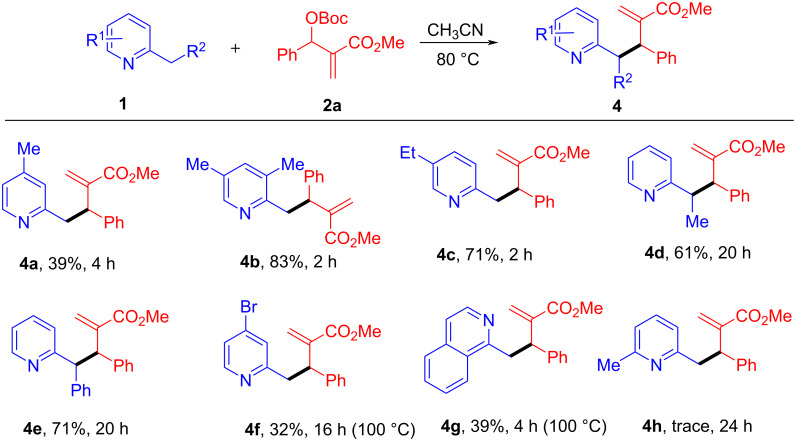
Scope of 2-alkylpyridine **1** with MBH carbonate **2a**. The reactions were performed using **1** (1.0 mmol, 2 equiv) and MBH carbonate **2** (0.5 mmol, 1 equiv) in CH_3_CN (2 mL) at 80 °C unless indicated otherwise. Yields are determined after silica gel column chromatography.

Subsequently, we set out to explore the scope of various 2-alkypyridines to react with MBH carbonate **2a** under the standard conditions and the results are shown in Scheme 4. Alkyl substituents at the 3 or 5-positions of pyridine were tolerated, giving the desired products in moderate to good yields (**4b** and **4c**, 83% and 71% yield, respectively). However, the reaction of 2,4-lutidine with **2a** led to complex results with several indistinct by-products formed, and only 39% yield of the desired product **4a** was isolated. The R^2^ group on the 2-alkylpyridines were then investigated. When R^2^ = Me or Ph, the reactions also proceeded well, providing products **4d** and **4e** in moderate yield (61% and 71% yield, respectively), although a longer reaction time (20 h) was needed. Similar to the reported results by Kim’s group [30], **4d** and **4e** were obtained as single isomers determining by ^1^H NMR spectra, but their relative stereo configurations (*syn*/*anti*) were not confirmed. 2-Methylpyridine bearing a 4-Br group and 2-methylisoquinoline were less reactive. When their reactions with **2a** were performed at higher temperature (100 °C) and for longer reaction time, the corresponding products **4f** and **4g** could be obtained although with low yields (32% and 39%, respectively), which was presumably due to their lower nucleophilicity. However, the reaction of **2a** with more sterically hindered 2,6-lutidine was very sluggish and only trace amount of product **4h** could be observed under the optimized conditions over 24 h.

## Conclusion

In summary, we have demonstrated a direct C(sp^3^)–H allylic alkylation reaction of 2-alkylpyridines with MBH carbonates with mild and simple operation. The process does not need either a base or a transition metal catalyst. The mechanism of this reaction was envisioned involving a tandem S_N_2’ type nucleophilic substitution followed by an aza-Cope rearrangement. The developed reaction provides a straightforward method for the synthesis of allylic functionalized 2-alkylpyridine derivatives, which could be useful as building blocks in organic and medicinal chemistry.

## Supporting Information

File 1 Experimental details, characterization data and copies of NMR spectra of new compounds.
