# Supplementary material for: Direct C(sp3)–H allylation of 2-alkylpyridines with Morita–Baylis–Hillman carbonates via a tandem nucleophilic substitution/aza-Cope rearrangement
Source: Beilstein J Org Chem. 2021 Oct 1;17:2505–10. doi: 10.3762/bjoc.17.167 (PMC8491709; doi:10.3762/bjoc.17.167)

BEILSTEIN JOURNAL OF ORGANIC CHEMISTRY

Supporting Information

for

Direct C(sp^3)–H allylation of 2-alkylpyridines with Morita–Baylis–Hillman carbonates via a tandem nucleophilic substitution/aza-Cope rearrangement

Siyu Wang, Lianyou Zheng, Shutao Wang, Shulin Ning, Zhuoqi Zhang and Jinbao Xiang

Beilstein J. Org. Chem. **2021,** *17,* 2505–2510. doi:10.3762/bjoc.17.167

Experimental details, characterization data and copies of NMR spectra of new compounds

License and Terms: This is a supporting information file under the terms of the Creative Commons Attribution License (https://creativecommons.org/licenses/by/4.0). Please note that the reuse, redistribution and reproduction in particular requires that the author(s) and source are credited and that individual graphics may be subject to special legal provisions.
The license is subject to the *Beilstein Journal of Organic Chemistry* terms and conditions: (https://www.beilstein-journals.org/bjoc/terms)

Table of Contents

page #

I. General experimental protocols S2

II. Preparation procedures and characterization data S3

III. References S12

VI. Copies of ^{1}H, ^{13}C NMR spectra S13

I. General experimental protocols

Acetonitrile was dried by refluxing with CaH_2 and then evaporation; other solvents and commercial reagents were used as received without additional purification. The Morita–Baylis–Hillman carbonates **2a–i** were prepared following the known procedure.[1-2] Flash column chromatography was performed using silica gel G (200–300 mesh). Reactions were monitored by thin-layer chromatography (TLC) (silica gel 60 F_{254}) and visualized using UV or $KMnO_4$. Melting points were measured on a SGW X-4 apparatus and were uncorrected.

^{1}H (and ^{13}C NMR) spectra were measured on a Varian spectrometer at 300 (or 75) MHz, respectively. Chemical shifts are reported in ppm (δ) with TMS as the internal standard and $CDCl_3$ or DMSO-d_6 as solvents. Multiplicities are indicated as the following: s = singlet, d = doublet, dd = doublet of doublets, t = triplet, q = quartet, m = multiplet and br s = broad singlet. Coupling constants (*J* values) are noted in Hertz. ^{13}C NMR spectra are determined with complete proton decoupling and reported in ppm.

II. Preparation procedures and characterization data.

General experimental procedure for the synthesis of compound ***3***

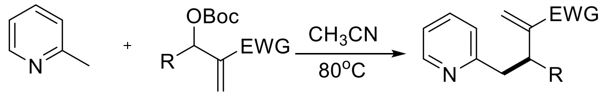

1a **2** **3**

2-Picoline (**1a**, 1.0 mmol) and Morita–Baylis–Hillman carbonates **2** (0.5 mmol) were dissolved in anhydrous CH_3CN (2.0 mL), and the mixture was stirred and heated in an oil bath at 80 °C under nitrogen atmosphere until the completion of the starting material **2** (monitored by TLC). After removing the solvent under vacuum, the residue was directly loaded on a silica gel column and purified by flash chromatography (eluting with petroleum ether/EtOAc), to provide the desired products **3**.

Methyl 2-methylene-3-phenyl-4-(pyridin-2-yl) butanoate (3a) [3]

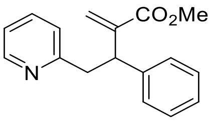

Eluting with petroleum ether/EtOAc = 5:1 (v/v). Colorless oil, yield 91%.

^{1}H NMR (300 MHz, Chloroform-*d*): δ 8.52– 8.49 (m, 1H), 7.47 (td, *J* = 7.8 Hz, 1.8 Hz, 1H), 7.25–7.11 (m, 5H), 7.05 (dd, *J* = 7.5 Hz, 4.8Hz, 1H), 6.93 (d, *J* = 7.8 Hz, 1H), 6.31(s, 1H), 5.76 (s, 1H), 4.51 (t, *J* = 7.8 Hz, 1H), 3.63 (s, 3H), 3.40 (dd, *J* = 13.8Hz, 7.5 Hz, 1H), 3.24 (dd, *J* = 13.5Hz, 8.4 Hz, 1H).

Methyl 3-(4-methoxyphenyl)-2-methylene-4-(pyridin-2-yl) butanoate (3b)

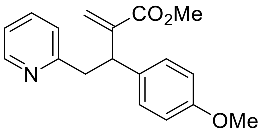

Eluting with petroleum ether/EtOAc = 8:1 (v/v). Pale yellow oil, yield 47%.

^{1}H NMR (300 MHz, Chloroform-*d*) δ 8.50 (ddd, *J* = 5.1Hz, 1.8Hz, 0.9Hz, 1H), 7.47 (td, *J* = 7.5Hz, 1.8Hz, 1H), 7.11–7.02 (m, 3H), 6.93(d, *J* = 7.8Hz, 1H), 6.78–6.73 (m, 2H), 6.28

(s, 1H), 5.74–5.72 (m, 1H), 4.45 (t, *J* = 8.1 Hz, 1H), 3.75 (s, 3H), 3.64 (s, 3H), 3.37 (dd, *J* = 13.8 Hz, 7.2 Hz, 1H), 3.19 (dd, *J* = 13.5 Hz, 8.7 Hz, 1H).

^{13}C NMR (75 MHz, Chloroform-*d*) *δ* 167.3, 159.8, 158.2, 149.3, 143.3, 136.1, 134.0, 129.1, 124.5, 123.6, 121.3, 113.7, 55.2, 51.9, 45.8, 43.3.

Methyl 2-methylene-3-(4-nitrophenyl)-4-(pyridin-2-yl) butanoate (3c)

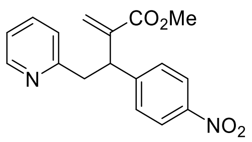

Eluting with petroleum ether/EtOAc = 5:1 (v/v). Yellow oil, yield 69%.

^{1}H NMR (300 MHz, Chloroform-*d*) *δ* 8.51 (ddd, *J* = 4.8, 1.8, 0.9 Hz, 1H), 8.06 (d, *J* = 8.7 Hz, 2H), 7.50 (td, *J* = 7.5 Hz, 1.8 Hz, 1H), 7.32 (d, *J* = 8.7 Hz, 2H), 7.09 (ddd, *J* = 7.8 Hz, 5.1 Hz, 1.2 Hz, 1H), 6.93 (dt, *J* = 7.8 Hz, 0.9 Hz, 1H), 6.41 (s, 1H), 5.87–5.85 (m, 1H), 4.65 (t, *J* = 7.8 Hz, 1H), 3.65 (s, 3H), 3.45 (A of AB, *J* = 13.8 Hz, 6.6 Hz, 1H), 3.22 (B of AB, *J* = 13.8 Hz, 9.3 Hz, 1H);

^{13}C NMR (75 MHz, Chloroform-*d*) *δ* 166.6, 158.5, 150.0, 149.6, 146.7, 141.8, 136.4, 129.0, 126.0, 123.6, 123.6, 121.7, 52.1, 46.5, 42.6.

Methyl 4-(3-(methoxycarbonyl)-1-(pyridin-2-yl) but-3-en-2-yl) benzoate (3d)

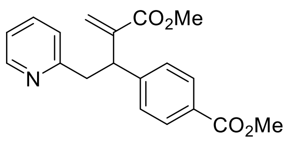

Eluting with petroleum ether/EtOAc = 10:1 (v/v). Colorless oil, yield 71%.

^{1}H NMR (300 MHz, Chloroform-*d*) *δ* 8.51 (ddd, *J* = 4.8, 1.8, 0.9 Hz, 1H), 7.88 (d, *J* = 8.4 Hz, 2H), 7.46 (td, *J* = 7.8, 1.8 Hz, 1H), 7.23 (d, *J* = 8.4 Hz, 2H), 7.06 (ddd, *J* = 7.5, 4.8, 0.9 Hz, 1H), 6.89 (d, *J* = 7.8 Hz, 1H), 6.36 (s, 1H), 5.80 (d, *J* = 0.6 Hz, 1H), 4.58 (t, *J* = 7.8 Hz, 1H), 3.87 (s, 3H), 3.63 (s, 3H), 3.41 (dd, *J* = 13.5 Hz, 6.9 Hz, 1H), 3.21 (dd, *J* = 13.5, 9.0 Hz, 1H);

^{13}C NMR (75 MHz, Chloroform-*d*) *δ* 167.1, 166.9, 159.1, 149.4, 147.5, 142.4, 136.2, 129.7, 128.5, 128.2, 125.5, 123.6, 121.5, 52.1, 52.0, 46.6, 42.9.

Methyl 3-(4-chlorophenyl)-2-methylene-4-(pyridin-2-yl) butanoate (3e)

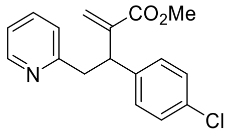

Eluted with petroleum ether/EtOAc = 14:1 (v/v). Pale yellow oil, yield 78%.

^{1}H NMR (300 MHz, Chloroform-*d*) *δ* 8.51 (ddd, *J* = 5.1Hz,1.8Hz,0.9Hz, 1H), 7.49 (td, *J* = 7.5Hz, 1.8Hz, 1H), 7.20–7.15 (m, 2H), 7.12–7.05 (m, 3H), 6.93 (d, *J* = 7.8Hz, 1H), 6.32 (t, *J* = 0.6Hz, 1H), 5.76 (dd, *J* = 1.2Hz, 0.6Hz,1H), 4.49 (t, *J* = 7.8Hz, 1H), 3.64 (s, 3H), 3.39 (dd, *J* = 13.8Hz, 6.9Hz, 1H), 3.19 (dd, *J* = 13.8Hz, 9.0Hz, 1H);

^{13}C NMR (75 MHz, Chloroform-*d*) *δ* 167.0, 159.2, 149.4, 142.7, 140.6, 136.2, 132.3, 129.5, 128.5, 125.1, 123.7, 121.4, 52.0, 46.0, 43.0.

Methyl 3-(4-bromophenyl)-2-methylene-4-(pyridin-2-yl) butanoate (3f)

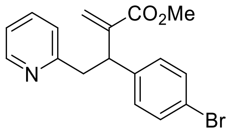

Eluting with petroleum ether/EtOAc = 14:1 (v/v). Colorless oil, yield 74%.

^{1}H NMR (300 MHz, Chloroform-*d*) *δ* 8.51 (ddd, *J* = 5.1, 1.5, 0.6 Hz, 1H), 7.49 (td, *J* = 7.8, 1.8 Hz, 1H), 7.36 – 7.30 (m, 2H), 7.10 – 7.00 (m, 3H), 6.92 (d, *J* = 7.8 Hz, 1H), 6.33 (s, 1H), 5.77 (s, 1H), 4.51 – 4.44 (m, 1H), 3.65 (s, 3H), 3.38 (dd, *J* = 13.5 Hz, 7.2 Hz, 1H), 3.17 (dd, *J* = 14.1 Hz, 9.3 Hz, 1H);

^{13}C NMR (75 MHz, Chloroform-*d*) *δ* 167.0, 159.2, 149.4, 142.6, 141.1, 136.3, 131.5, 129.9, 125.2, 123.7, 121.5, 120.5, 52.0, 46.1, 43.0.

Methyl 3-(4-fluorophenyl)-2-methylene-4-(pyridin-2-yl) butanoate (3g)

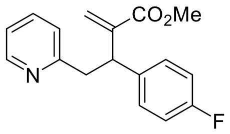

Eluting with petroleum ether/EtOAc = 14:1 (v/v). Colorless oil, yield 69%.

^{1}H NMR (300 MHz, Chloroform-*d*) *δ* 8.53 – 8.49 (m, 1H), 7.48 (td, *J* = 7.5 Hz, 1.8 Hz, 1H), 7.15 – 7.03 (m, 3H), 6.94 – 6.86 (m, 3H), 6.32 (s, 1H), 5.76 (s, 1H), 4.53 – 4.45 (m, 1H), 3.65 (s, 3H), 3.39 (dd, *J* = 13.5 Hz, 6.6 Hz, 1H), 3.18 (dd, *J* = 13.5 Hz, 9.0 Hz, 1H); **^{13}C NMR** (75 MHz, Chloroform-*d*) *δ* 167.1, 161.5 (d, *J* = 243 Hz), 159.3, 149.4, 142.9, 137.6 (d, *J* = 3 Hz), 136.1, 129.6 (d, *J* = 7.5 Hz), 124.8, 123.6, 121.3, 115.1 (d, *J* = 21 Hz), 51.9, 45.9, 43.2.

Methyl 2-methylene-3-(naphthalen-2-yl)-4-(pyridin-2-yl) butanoate (3h)

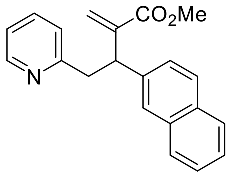

Eluting with petroleum ether/EtOAc = 14:1 (v/v). Colorless oil, yield 65%.
^{1}H NMR (300 MHz, Chloroform-*d*) *δ* 8.51 (ddd, *J* = 5.1 Hz, 1.8 Hz, 0.9 Hz, 1H), 7.79 – 7.72 (m, 2H), 7.71 (d, *J* = 8.4 Hz, 1H), 7.62 (dd, *J* = 1.2 Hz, 0.3 Hz, 1H), 7.46 – 7.37 (m, 3H), 7.31 (dd, *J* = 8.7Hz, 1.8 Hz, 1H), 7.04 (ddd, *J* = 7.5 Hz, 4.8 Hz, 1.2 Hz, 1H), 6.93 (dt, *J* = 7.8 Hz, 0.9 Hz, 1H), 6.36 (s, 1H), 5.83 – 5.81 (m, 1H), 4.70 (t, *J* = 7.8 Hz, 1H), 3.62 (s, 3H), 3.48 (dd, *J* = 13.8 Hz, 7.2 Hz, 1H), 3.33 (dd, *J* = 13.5 Hz, 8.4 Hz, 1H); **^{13}C NMR** (75 MHz, Chloroform-*d*) *δ* 167.3, 159.6, 149.4, 143.0, 139.6, 136.2, 133.5, 132.4, 128.0, 127.9, 127.6, 126.7, 125.9, 125.6, 125.2, 125.2, 123.6, 121.3, 52.0, 46.6, 43.2.

Methyl 2-methylene-4-(pyridin-2-yl)-3-(thiophen-2-yl) butanoate (3i)

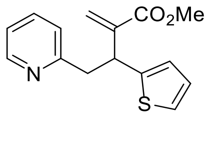

Eluted with petroleum ether/EtOAc = 16:1 (v/v). Pale yellow oil, yield 26%.
^{1}H NMR (300 MHz, Chloroform-*d*) *δ* 8.55 – 8.50 (m, 1H), 7.52 (td, *J* = 7.8 Hz, 1.8 Hz, 1H), 7.13 – 7.06 (m, 2H), 7.04 (d, *J* = 7.8 Hz, 1H), 6.86 (dd, *J* = 5.1 Hz, 3.3 Hz, 1H), 6.82 – 6.78 (m, 1H), 6.28 (s, 1H), 5.75 (s, 1H), 4.81 (t, *J* = 8.1 Hz, 1H), 3.70 (s, 3H), 3.45 (dd, *J* = 13.8 Hz, 7.5 Hz, 1H), 3.35 (dd, *J* = 13.5 Hz, 8.4 Hz, 1H);

^{13}C NMR (75 MHz, Chloroform-*d*) *δ* 167.0, 159.2, 149.4, 145.9, 142.8, 136.2, 126.7, 125.7, 125.0, 123.8, 123.7, 121.5, 52.1, 44.1, 42.1.

Methyl 2-methylene-4-(pyridin-2-yl) butanoate (3j)

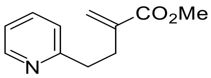

Eluting with petroleum ether/EtOAc = 14:1 (v/v). Colorless oil, yield 84%.
^{1}H NMR (300 MHz, Chloroform-*d*) *δ* 8.54 (ddd, *J* = 4.8 Hz, 1.5 Hz, 0.9 Hz, 1H), 7.59 (td, *J* = 7.8 Hz, 1.8 Hz, 1H), 7.17 – 7.08 (m, 2H), 6.17 – 6.14 (m, 1H), 5.54 (q, *J* = 1.5 Hz, 1H), 3.77 (s, 3H), 3.01 – 2.95 (m, 2H), 2.80 – 2.71 (m, 2H);
^{13}C NMR (75 MHz, Chloroform-*d*) *δ* 167.6, 161.0, 149.4, 139.7, 136.4, 125.8, 123.1, 121.3, 52.0, 37.2, 32.1.

Methyl 2-methylene-5-phenyl-3-(pyridin-2-ylmethyl) pentanoate (3k)

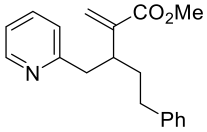

Eluting with petroleum ether/EtOAc = 16:1 (v/v). Colorless oil, yield 38%.
^{1}H NMR (300 MHz, Chloroform-*d*) *δ* 8.52 (ddd, *J* = 4.8 Hz, 1.8 Hz, 0.9 Hz, 1H), 7.55 (td, *J* = 7.5 Hz, 1.8 Hz, 1H), 7.26 – 7.20 (m, 2H), 7.19 – 7.04 (m, 5H), 6.20 (d, *J* = 0.9 Hz, 1H), 5.54 – 5.52 (m, 1H), 3.73 (s, 3H), 3.24 – 3.09 (m, 1H), 3.09 – 2.96 (m, 2H), 2.67 – 2.44 (m, 2H), 2.02 – 1.77 (m, 2H);
^{13}C NMR (75 MHz, Chloroform-*d*) *δ* 167.6, 160.3, 149.3, 142.6, 142.3, 136.2, 128.4, 128.4, 126.0, 125.8, 123.7, 121.2, 51.9, 43.3, 42.3, 35.4, 33.7.

tert-Butyl 2-methylene-3-phenyl-4-(pyridin-2-yl) butanoate (3l)

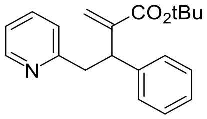

Eluting with petroleum ether/EtOAc = 17:1 (v/v). Colorless oil, yield 39%.

^{1}H NMR (300 MHz, Chloroform-*d*) *δ* 8.51 (ddd, *J* = 5.1 Hz, 1.8 Hz, 0.9 Hz, 1H), 7.44 (td, *J* = 7.8 Hz, 1.8 Hz, 1H), 7.23 – 7.01 (m, 6H), 6.87 (dt, *J* = 7.8 Hz, 0.9 Hz, 1H), 6.25 (t, *J* = 0.9 Hz, 1H), 5.65 (t, *J* = 1.2 Hz, 1H), 4.41 (t, *J* = 7.8 Hz, 1H),3.39 (dd, *J* = 13.5, 6.9 Hz, 1H), 3.16 (dd, *J* = 13.5, 9.0 Hz, 1H), 1.30 (s, 9H);
^{13}C NMR (75 MHz, Chloroform-*d*) *δ* 166.1, 159.8, 149.3, 144.8, 142.4, 136.0, 128.2, 126.4, 123.7, 121.2, 80.8, 46.7, 43.3, 28.0.

Benzyl 2-methylene-3-phenyl-4-(pyridin-2-yl) butanoate (3m)

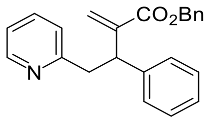

Eluting with petroleum ether/EtOAc = 16:1 (v/v). Colorless oil, yield 48%.
^{1}H NMR (300 MHz, Chloroform-*d*) *δ* 8.49 (ddd, *J* = 4.8 Hz, 1.5 Hz, 0.6 Hz, 1H), 7.44 (td, *J* = 7.5, 1.8 Hz, 1H), 7.32 – 7.26 (m, 3H), 7.24 – 7.11 (m, 7H), 7.04 (ddd, *J* = 7.2 Hz, 4.8 Hz, 0.9 Hz, 1H), 6.89 (d, *J* = 7.8 Hz, 1H), 6.37 (s, 1H), 5.80 – 5.77 (m, 1H), 5.11 (A of AB, *J* = 12.6 Hz, 1H), 5.03 (B of AB, *J* = 12.6 Hz, 1H), 4.52 (t, *J* = 7.8 Hz, 1H), 3.40 (dd, *J* = 13.8 Hz, 7.2 Hz, 1H), 3.22 (dd, *J* = 13.8 Hz, 8.7 Hz, 1H);
^{13}C NMR (75 MHz, Chloroform-*d*) *δ* 166.6, 159.6, 149.3, 142.9, 142.0, 136.1, 136.0, 128.5, 128.4, 128.2, 128.1, 128.1, 126.6, 125.3, 123.6, 121.3, 66.6, 46.6, 43.2.

2-Methylene-3-phenyl-4-(pyridin-2-yl) butanenitrile (3n) [3]

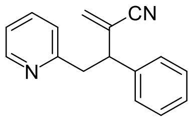

Eluting with petroleum ether/EtOAc = 10:1 (v/v). Colorless oil, yield 53%.
^{1}H NMR (300 MHz, Chloroform-*d*) δ 8.55 (ddd, *J* = 4.8 Hz, 1.8 Hz, 0.9 Hz, 1H), 7.54 (td, *J* = 7.8 Hz, 1.8 Hz, 1H), 7.36 – 7.21 (m, 5H), 7.14 – 7.05 (m, 2H), 5.81 (s, 1H), 5.74 (d, *J* = 0.9 Hz, 1H), 4.23 (t, *J* = 7.8 Hz, 1H), 3.43 (dd, *J* = 13.8 Hz, 8.4 Hz, 1H), 3.31 (dd, *J* = 13.8 Hz, 7.2 Hz, 1H).

General experimental procedure for the synthesis of compounds 4

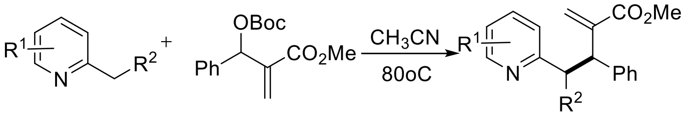

1 **2a** **4**

Similar to the procedure for the synthesis of compounds **3**, the picoline derivative **1** (1.0 mmol) and Morita–Baylis–Hillman carbonate **2a** (0.5 mmol) were dissolved in anhydrous CH_3CN (2.0 mL) and then stirred and heated in an oil bath at 80 °C (or 100 °C as indicated) until the completion of **2a**. After removing the solvent under vacuum, the residue wad directly purified by flash chromatography on silica gel column (eluting with petroleum ether/EtOAc), to provide the desired products **4**.

Methyl 2-methylene-4-(4-methylpyridin-2-yl)-3-phenylbutanoate (4a)

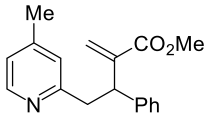

Eluting with petroleum ether/EtOAc = 5:1 (v/v). Colorless oil, yield 39%.

^{1}H NMR (300 MHz, Chloroform-*d*) *δ* 8.35 (d, *J* = 5.1 Hz, 1H), 7.24–7.13 (m, 5H), 6.89 (d, *J* = 4.8 Hz, 1H), 6.79 (s, 1H), 6.31(s, 1H), 5.77 (s, 1H), 4.51 (t, *J* = 7.8 Hz, 1H), 3.64 (s, 3H), 3.36 (A of AB, *J* = 13.8, 7.5 Hz, 1H), 3.20 (B of AB, *J* = 13.8, 8.4 Hz, 1H), 2.24 (s, 3H);

^{13}C NMR (75 MHz, Chloroform-*d*) *δ* 167.4, 159.3, 148.7,147.6, 143.1, 142.2, 128.4, 128.2, 126.6, 125.0, 124.6, 122.5, 51.9, 46.6, 42.9, 21.1.

Methyl 4-(3,5-dimethylpyridin-2-yl)-2-methylene-3-phenylbutanoate (4b)

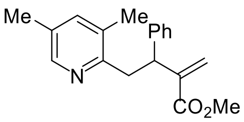

Eluting with petroleum ether/EtOAc = 5:1 (v/v). Pale yellow oil, yield 83%.

^{1}H NMR (300 MHz, Chloroform-*d*) *δ* 8.18 (s, 1H), 7.23–7.10 (m, 6H), 6.34 (s, 1H), 5.77 (s, 1H), 4.56 (t, *J* = 7.8 Hz, 1H), 3.61 (s, 3H), 3.31–3.14 (m, 2H), 2.23 (s, 3H), 1.99 (s, 3H);

^{13}**C NMR** (75 MHz, Chloroform-*d*) *δ* 167.4, 154.8, 147.1, 143.4, 142.4, 138.3, 131.1, 130.5, 128.2, 128.1, 126.5, 124.6, 51.9, 45.9, 39.6, 18.6, 18.0.

Methyl 4-(5-ethylpyridin-2-yl)-2-methylene-3-phenylbutanoate (4c)[3]

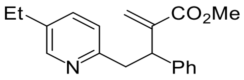

Eluting with petroleum ether/EtOAc = 10:1 (v/v). Colorless oil, yield 71%.

^{1}**H NMR** (300 MHz, Chloroform-*d*) *δ* 8.34 (d, *J* = 2.1Hz, 1H), 7.30 (dd, *J* = 8.1Hz, 2.4Hz, 1H), 7.26–7.11 (m, 5H), 6.86 (d, *J* = 7.8 Hz, 1H), 6.31 (s, 1H), 5.76 (t, *J* = 0.9 Hz,1H), 4.50 (t, *J* = 8.1 Hz, 1H), 3.63 (s, 3H), 3.36 (A of AB, *J* = 13.8, 7.5 Hz, 1H), 3.20 (B of AB, *J* = 13.8, 8.4 Hz, 1H), 2.58 (q, *J* = 7.8 Hz, 2H), 1.21 (t, *J* = 7.8 Hz, 3H);

^{13}**C NMR** (75 MHz, Chloroform-*d*) *δ* 167.3, 156.8, 148.9, 143.0, 142.2, 136.7, 135.6, 128.3, 128.2, 126.5, 125.0, 123.1, 51.9, 46.5, 42.7, 25.8, 15.3.

Methyl 2-methylene-3-phenyl-4-(pyridin-2-yl) pentanoate (4d)

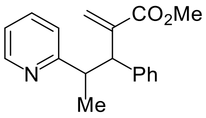

Eluting with petroleum ether/EtOAc = 10:1 (v/v). Colorless oil, yield 61%.

^{1}**H NMR** (300 MHz, Chloroform-*d*) *δ* 8.56–8.52 (m, 1H), 7.65 (t, *J* = 7.8Hz, 1H), 7.41–7.29 (m, 5H), 7.26–7.20 (m, 1H), 7.14 (t, *J* = 6.9Hz, 1H), 6.07 (s, 1H), 5.76 (s, 1H), 4.38 (d, *J* = 12.0Hz, 1H), 3.71–3.61(m, 1H), 3.58 (s, 3H), 1.09 (d, *J* = 6.9 Hz, 3H);

^{13}**C NMR** (75 MHz, Chloroform-*d*) *δ* 167.3, 165.1, 149.2, 142.5, 141.6, 136.7, 128.9, 128.5, 126.8, 125.5, 122.2, 121.4, 51.9, 51.9, 45.5, 21.2.

Methyl 2-methylene-3,4-diphenyl-4-(pyridin-2-yl) butanoate (4e)[3]

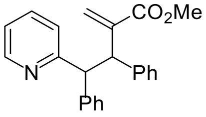

Eluting with petroleum ether/EtOAc = 10:1 (v/v). White solid, m. p.: 165-167°C, yield 71%.

^{1}H NMR (300 MHz, Chloroform-*d*) *δ* 8.54 (ddd, *J* = 4.8 Hz, 1.8 Hz, 0.9 Hz, 1H), 7.56 (td, *J* = 7.8 Hz, 2.1 Hz, 1H), 7.30 (d, *J* = 7.8 Hz, 1H), 7.21-6.95 (m, 11H), 6.16 (s, 1H), 5.73 (s, 1H), 5.06 (d, *J* = 12.6 Hz, 1H), 4.73 (d, *J* = 12.6 Hz, 1H), 3.63 (s, 3H).

Methyl 4-(4-bromopyridin-2-yl)-2-methylene-3-phenylbutanoate(4f)

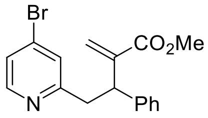

The reaction was performed at 100 ^{o}C for 16h. Eluting with petroleum ether/EtOAc = 10:1 (v/v). Colorless oil, yield 32%.
^{1}H NMR (300 MHz, Chloroform-*d*) *δ* 8.31 (d, *J* = 5.1 Hz, 1H), 7.26–7.13 (m, 7H), 6.32 (s, 1H), 5.73 (s, 1H), 4.49 (t, *J* = 7.8 Hz, 1H), 3.65 (s, 3H), 3.38 (dd, *J* = 13.8Hz, 7.5 Hz, 1H), 3.20 (dd, *J* = 13.8 Hz, 8.7 Hz, 1H);
^{13}C NMR (75 MHz, Chloroform-*d*) *δ* 167.2, 161.3, 150.0, 142.7, 141.6, 132.9, 128.5, 128.1, 127.0, 126.8, 125.2, 124.8, 52.0, 46.4, 42.8.

Methyl 4-(isoquinolin-1-yl)-2-methylene-3-phenylbutanoate (4g)

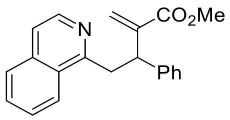

The reaction was performed at 100 ^{o}C for 4h. Eluting with petroleum ether/EtOAc = 10:1 (v/v). Pale yellow oil, yield 39%.
^{1}H NMR (300 MHz, Chloroform-*d*) *δ* 8.38 (d, *J* = 5.7 Hz, 1H), 8.13 (d, *J* = 8.4 Hz, 1H), 7.79 – 7.76 (m, 1H), 7.66 –7.60 (m, 1H), 7.57-7.51 (m, 1H), 7.46 (d, *J* = 5.7 Hz, 1H), 7.22 – 7.11 (m, 5H), 6.31 (s, 1H), 5.78 – 5.77 (m, 1H), 4.79 (td, *J* = 6.9 Hz, 0.9 Hz, 1H), 3.91 (dd, *J* = 14.1Hz, 7.5 Hz, 1H), 3.76 (dd, *J* = 14.1, 7.8 Hz, 1H), 3.62 (s, 3H).
^{13}C NMR (75 MHz, Chloroform-*d*) *δ* 167.4, 159.3, 143.1, 142.2, 141.8, 136.2, 129.8, 128.4, 128.1, 127.5, 127.1, 126.6, 125.2, 125.1, 119.5, 51.9, 46.2, 39.8.

III. References

1、Yang, X. -H.; Li, J. -P.; Wang, D. -C.; Xie, M. -S.; Qu, G. -R.; Guo, H. -M. *Chem.Commun.* **2019,** *55,* 9144-9147. doi: 10.1039/c9cc04542b

2、Pautigny, C.; Jeulin, S.; Ayad, T.; Zhang, Z.; Genet, J.-P.; Ratovelomanana-Vidal, V. *Adv. Synth. Catal.* **2008,** *350,* 2525-2532. doi: 10.1002/adsc.200800504

3、Lee, H. S.; Lee, S.; Kim, S. H.; Kim, J. N. *Tetrahedron Lett.* **2011,** *52,* 5039-5042. doi: 10.1016/j.tetlet.2011.07.101

VI. Copies of ^{1}H, ^{13}C NMR spectra

^{1}H NMR spectra of compound 3a

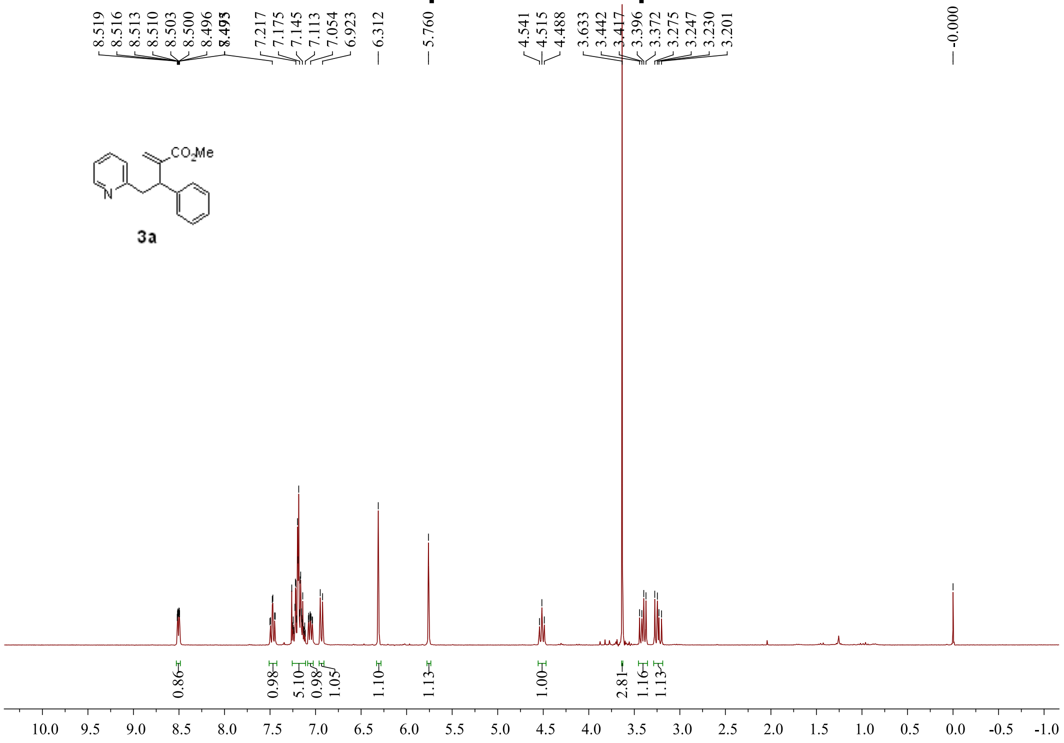

^{1}H NMR spectra of compound 3b

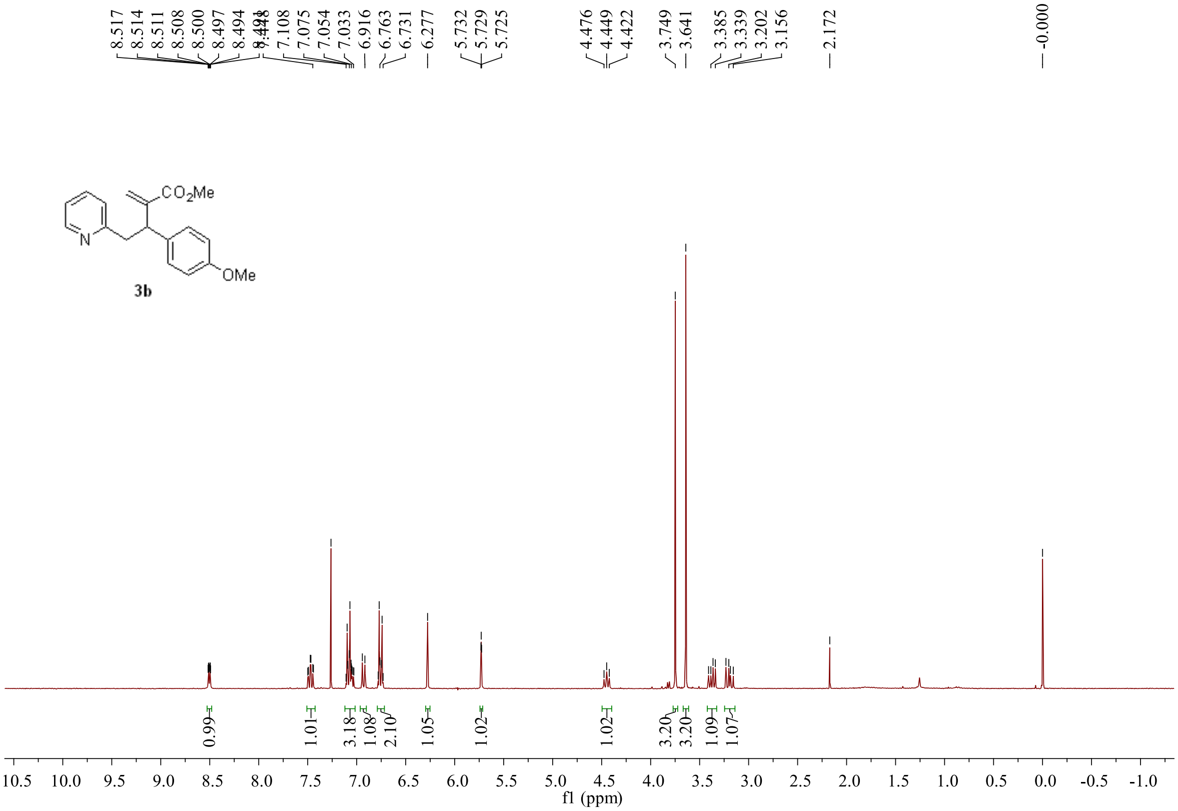

^{13}C NMR spectra of compound 3b

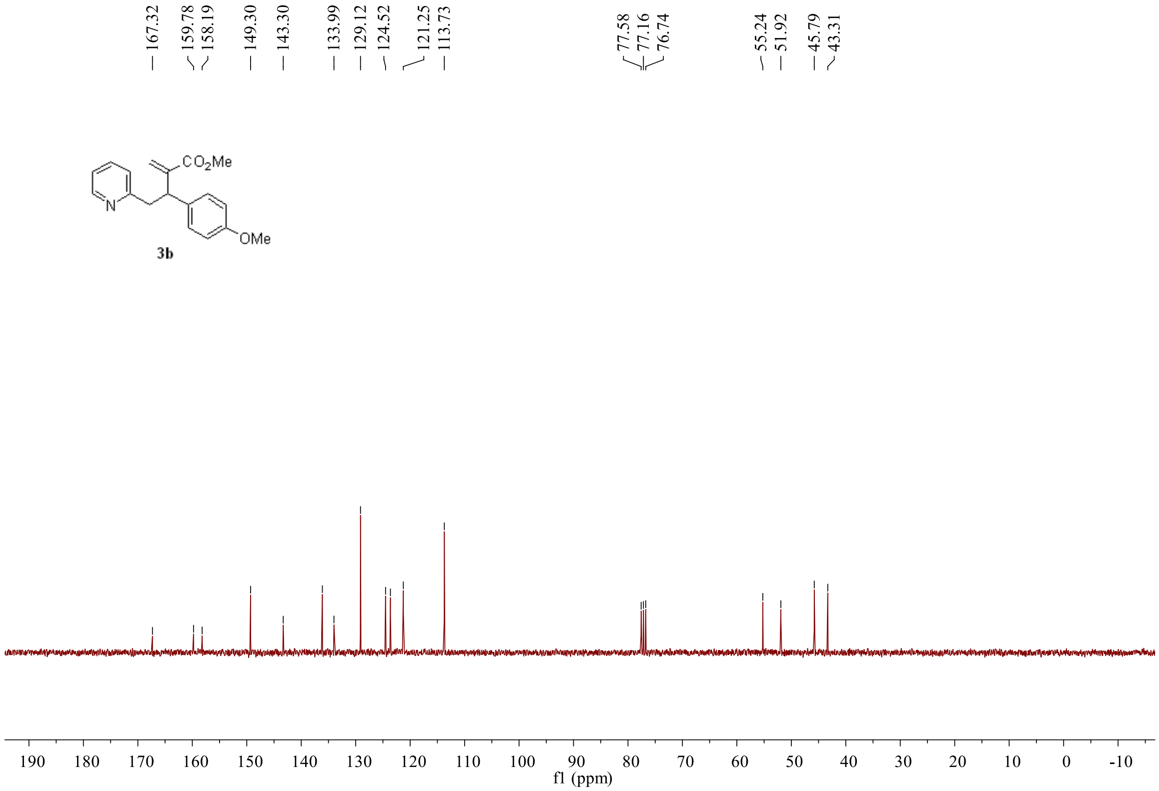

^{1}H NMR spectra of compound 3c

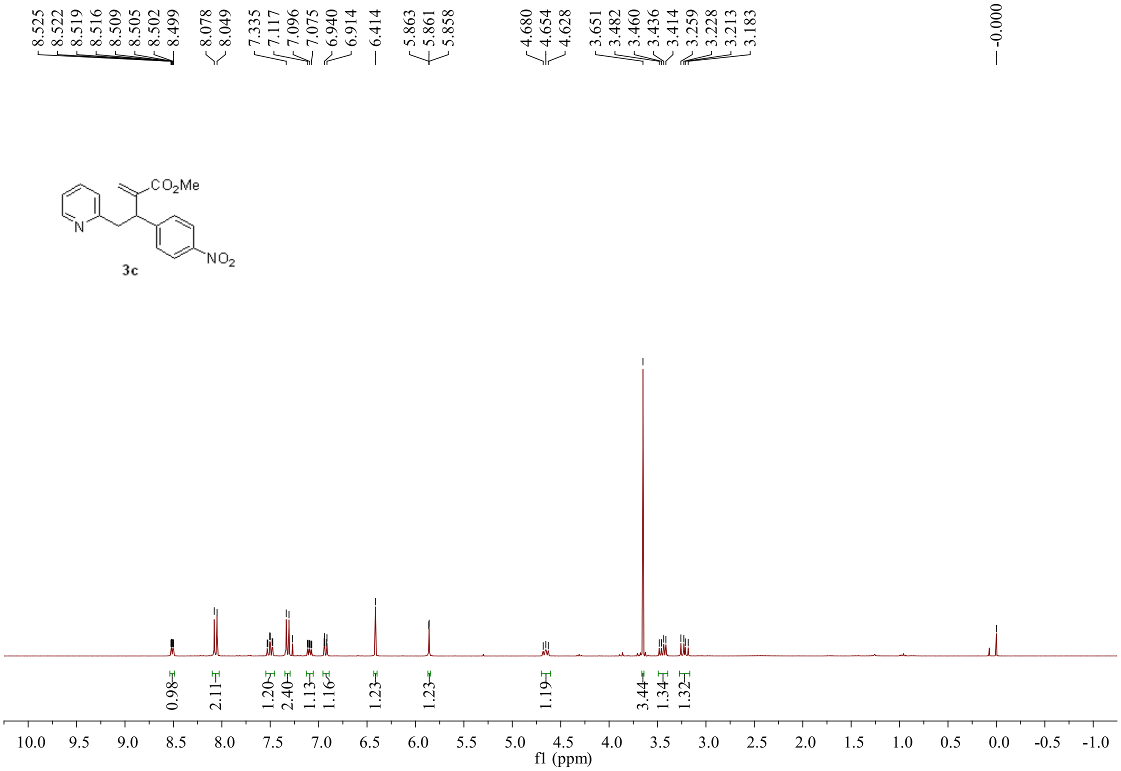

^{13}C NMR spectra of compound 3c

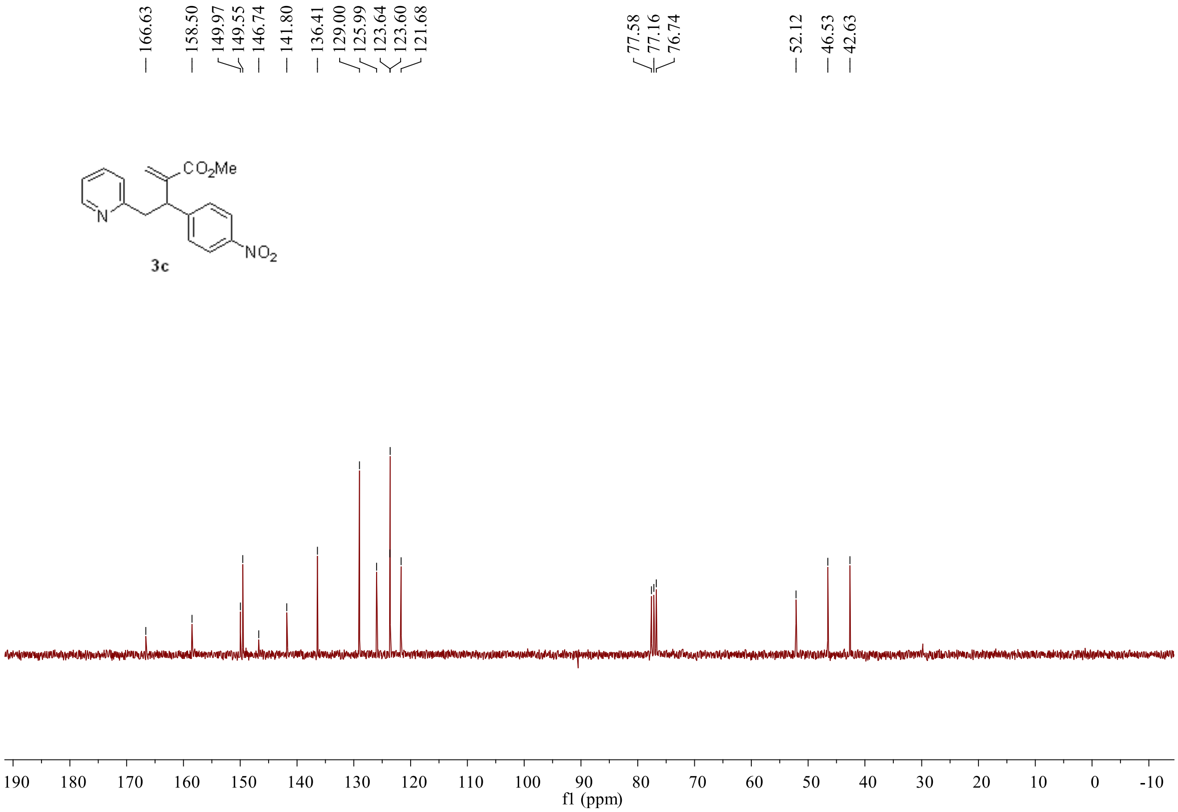

^{1}H NMR spectra of compound 3d

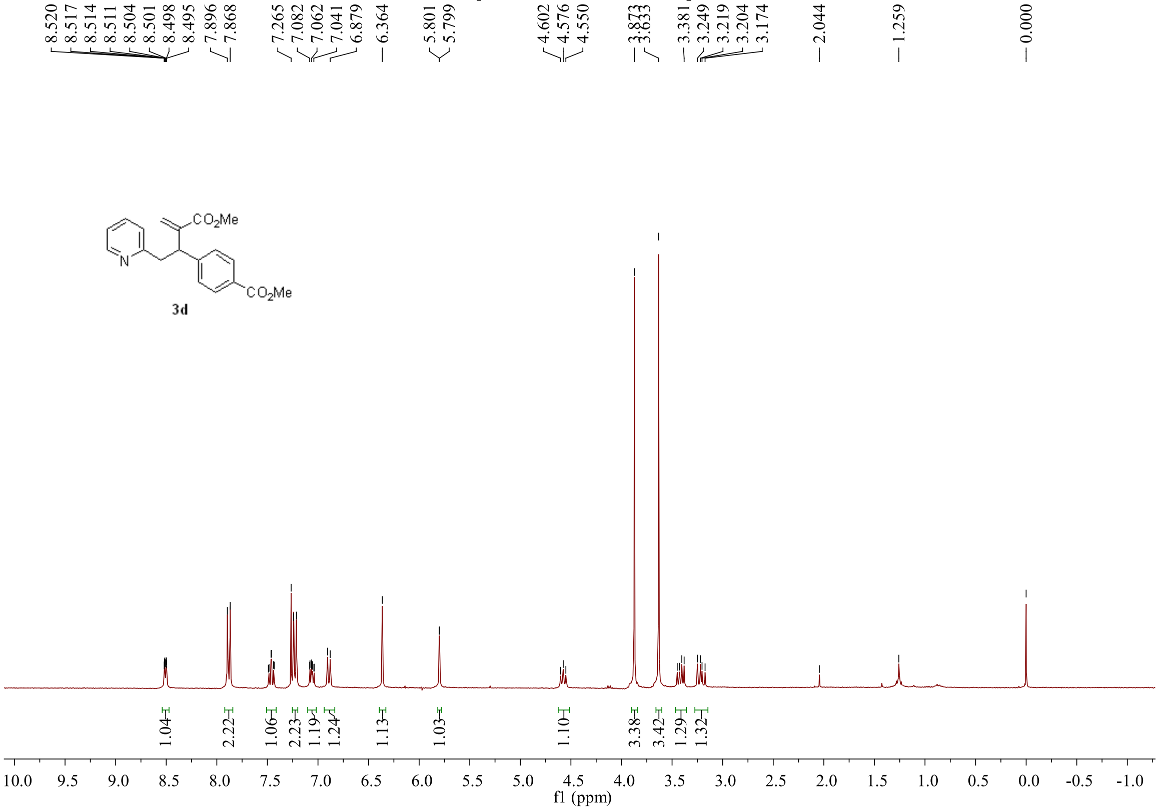

^{13}C NMR spectra of compound 3d

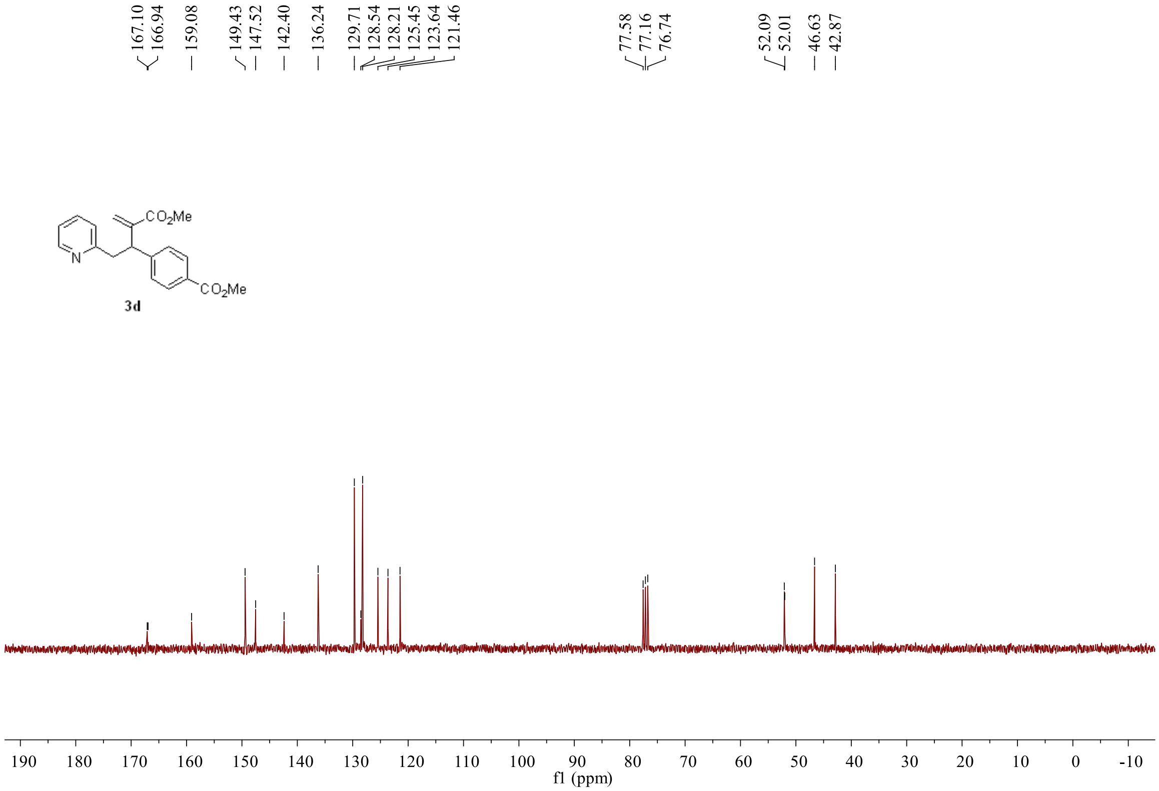

^{1}H NMR spectra of compound 3e

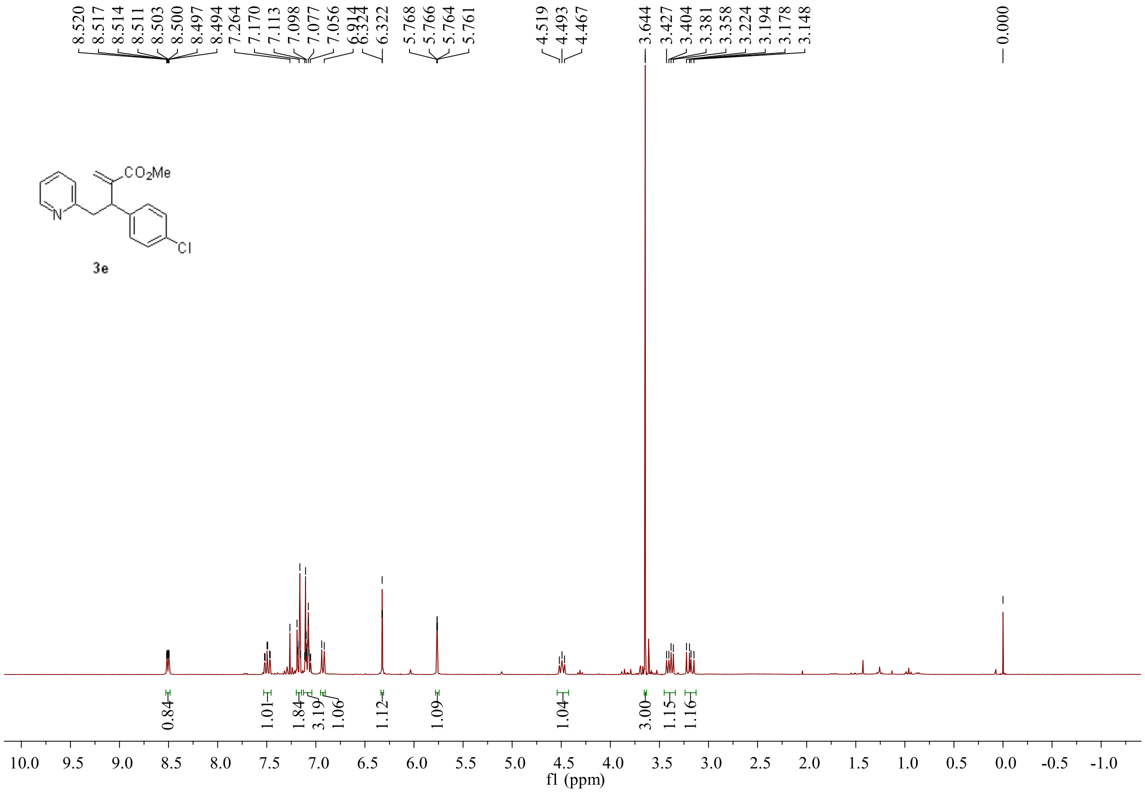

^{13}C NMR spectra of compound 3e

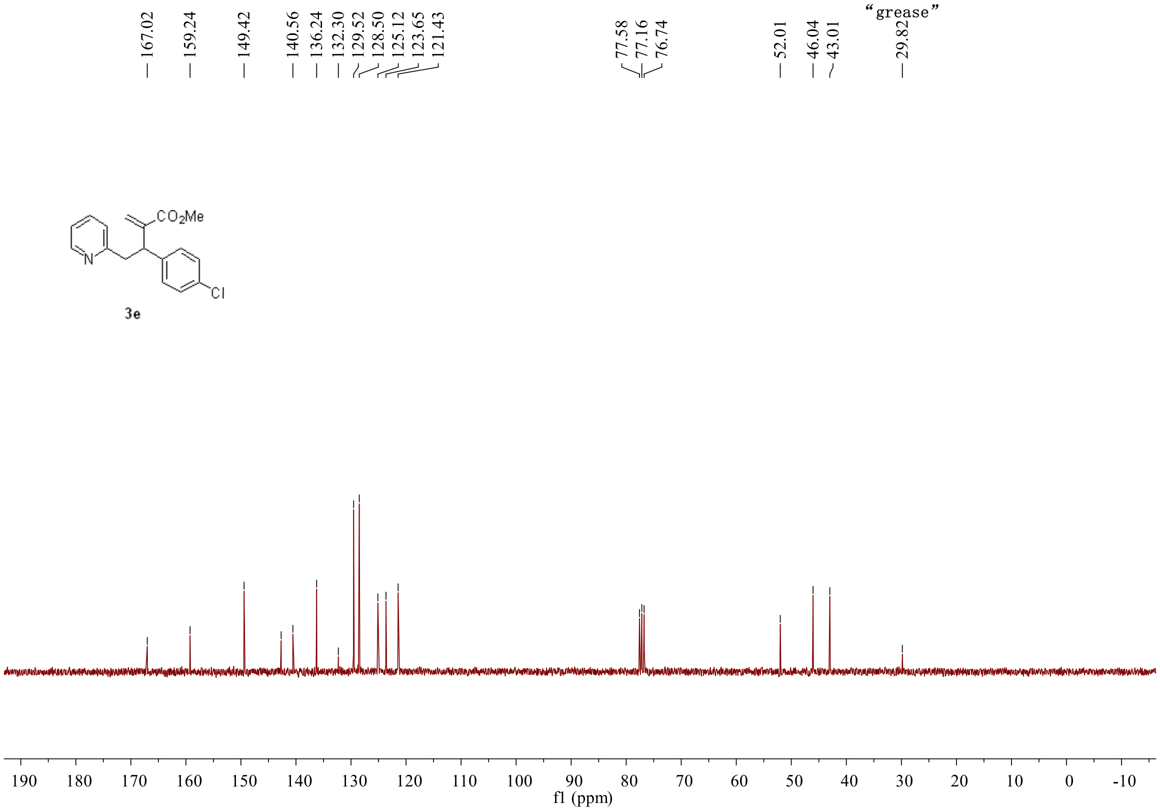

^{1}H NMR spectra of compound 3f

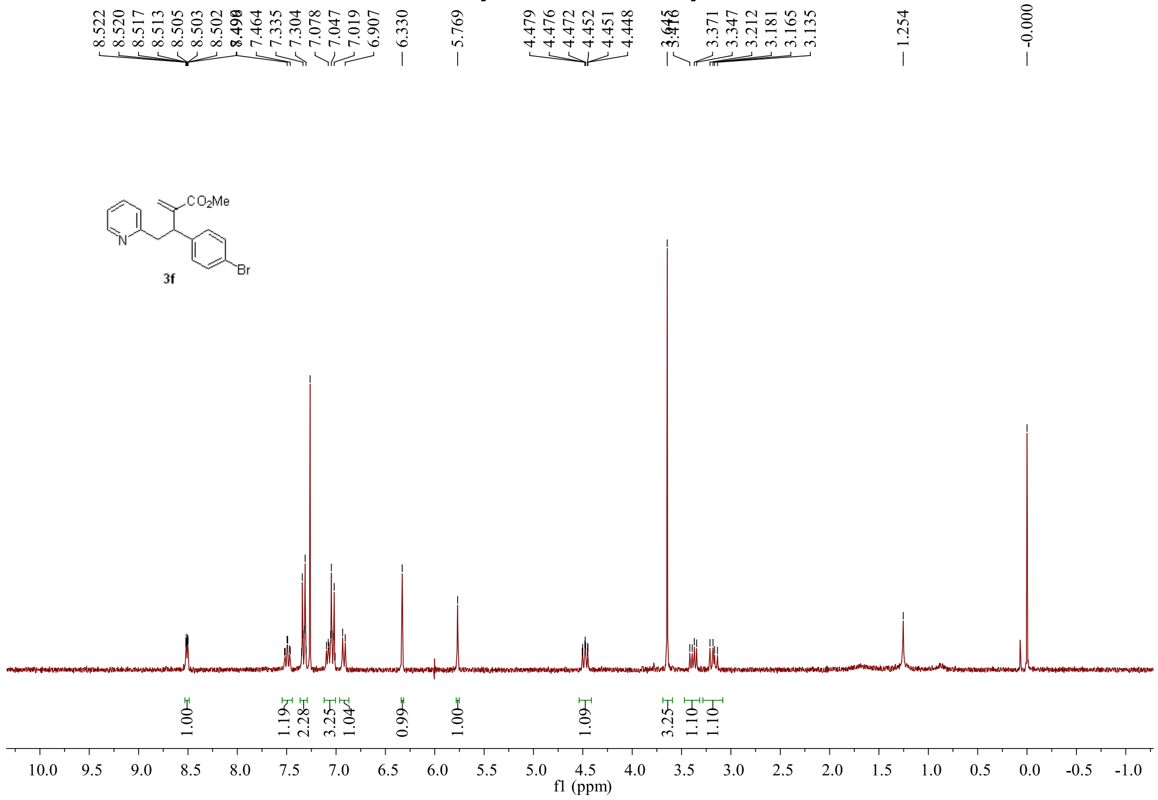

^{13}C NMR spectra of compound 3f

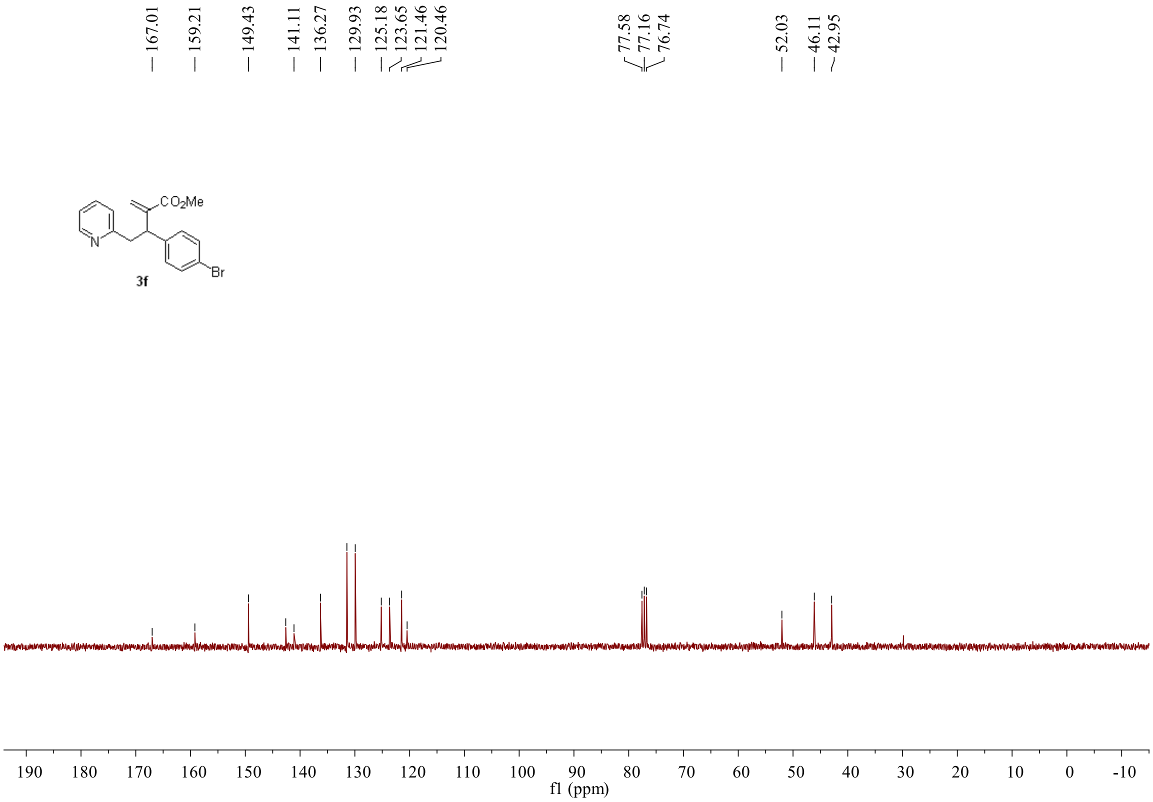

^{1}H NMR spectra of compound 3g

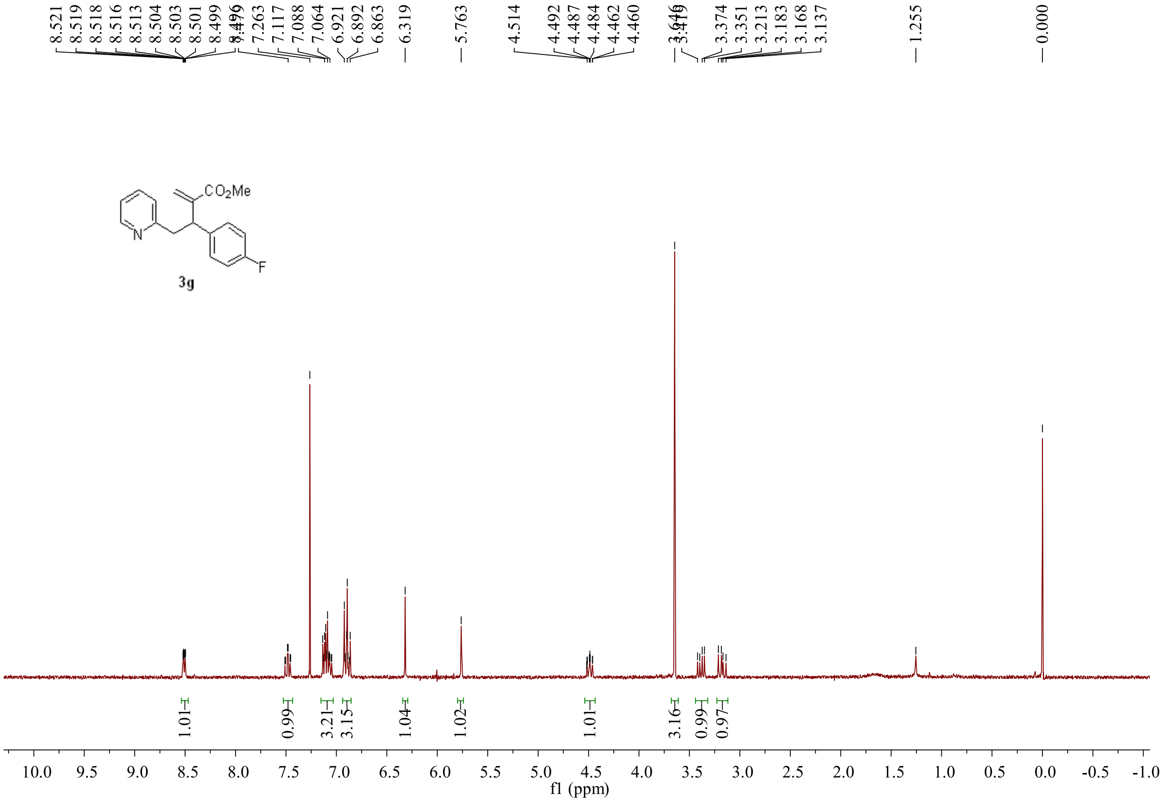

^{13}C NMR spectra of compound 3g

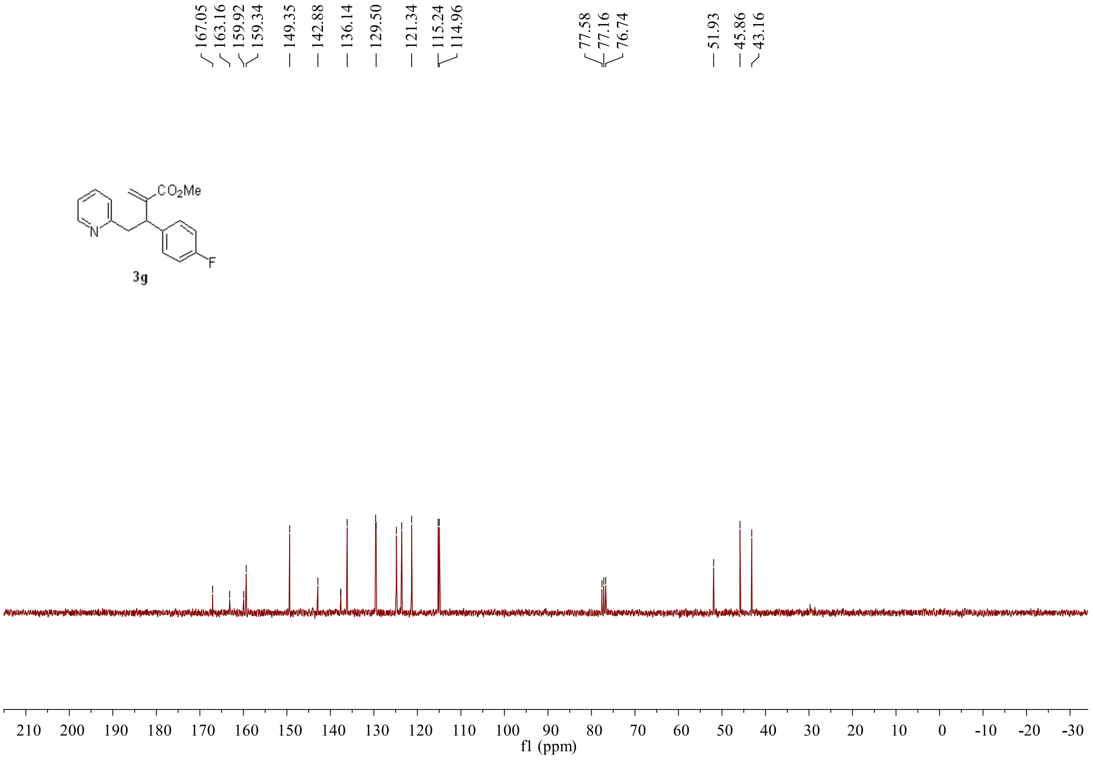

^{1}H NMR spectra of compound 3h

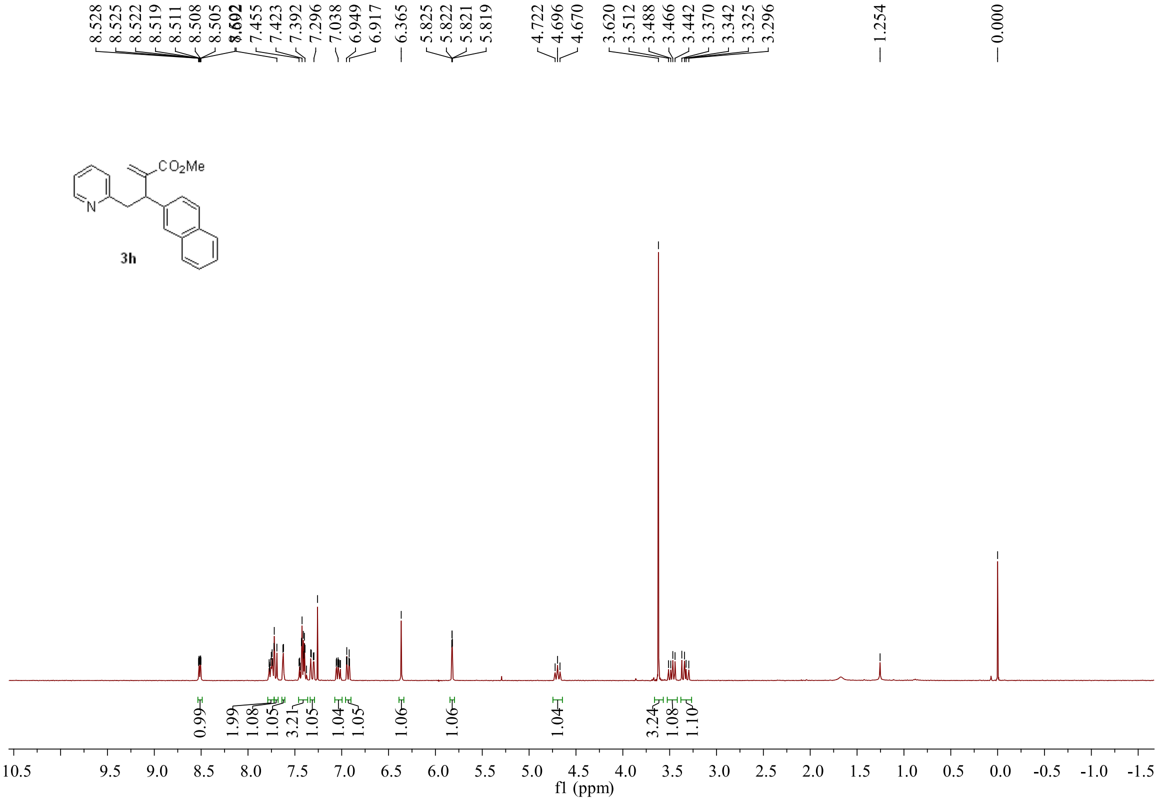

^{13}C NMR spectra of compound 3h

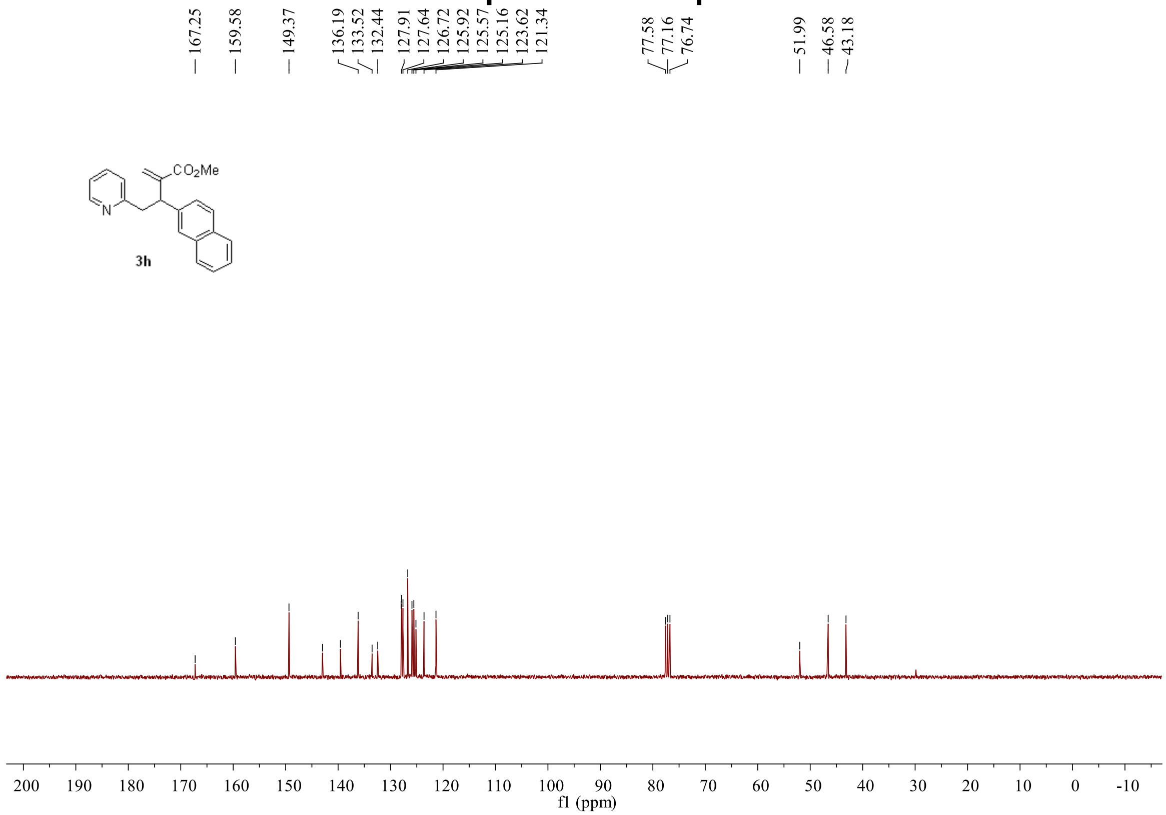

^{1}H NMR spectra of compound 3i

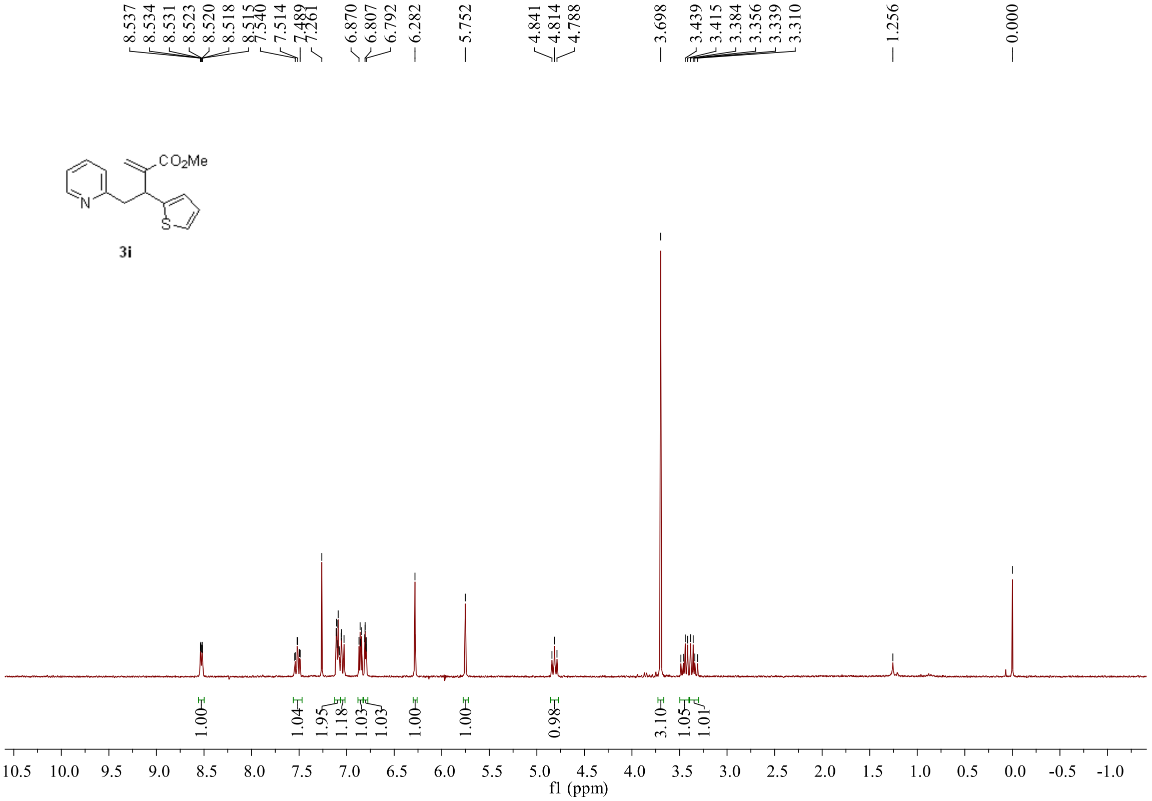

^{13}C NMR spectra of compound 3i

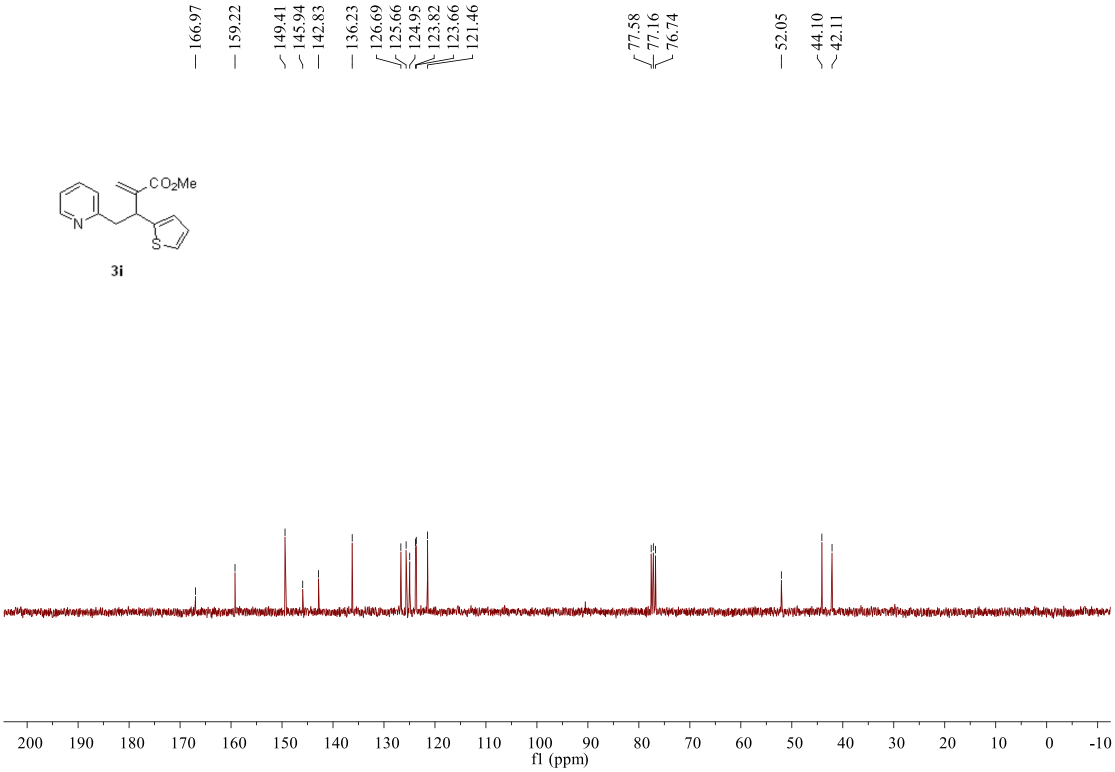

^{1}H NMR spectra of compound 3j

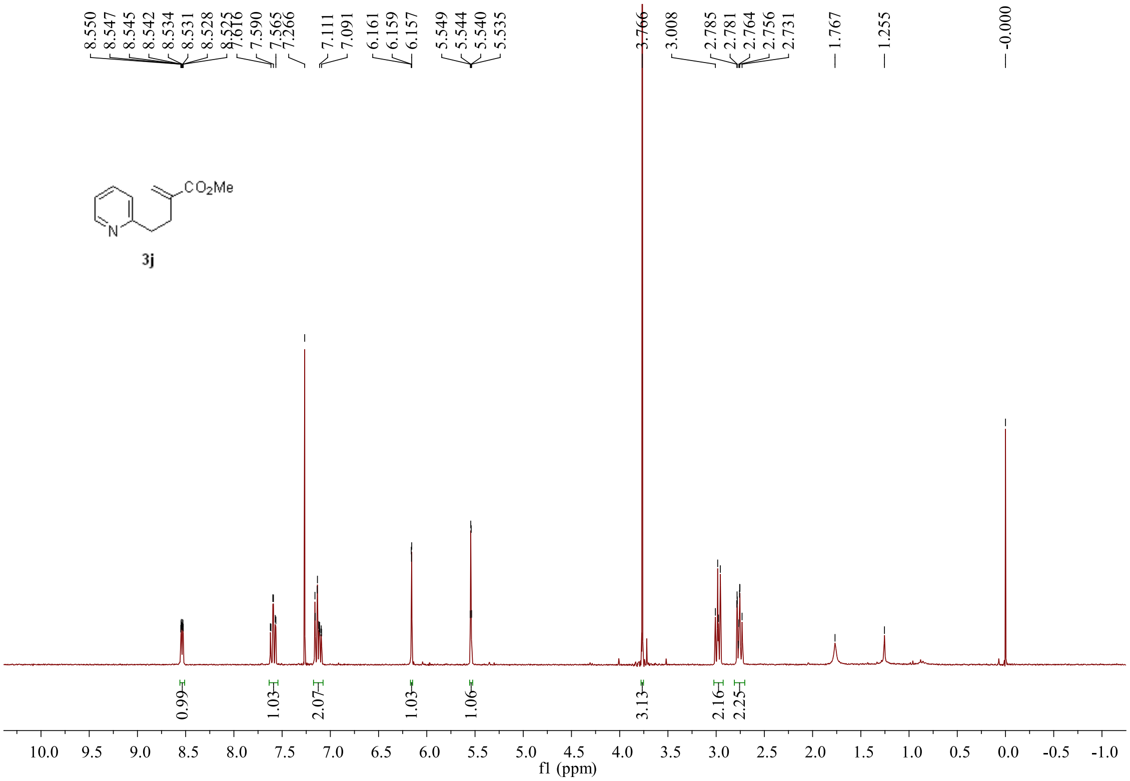

^{13}C NMR spectra of compound 3j

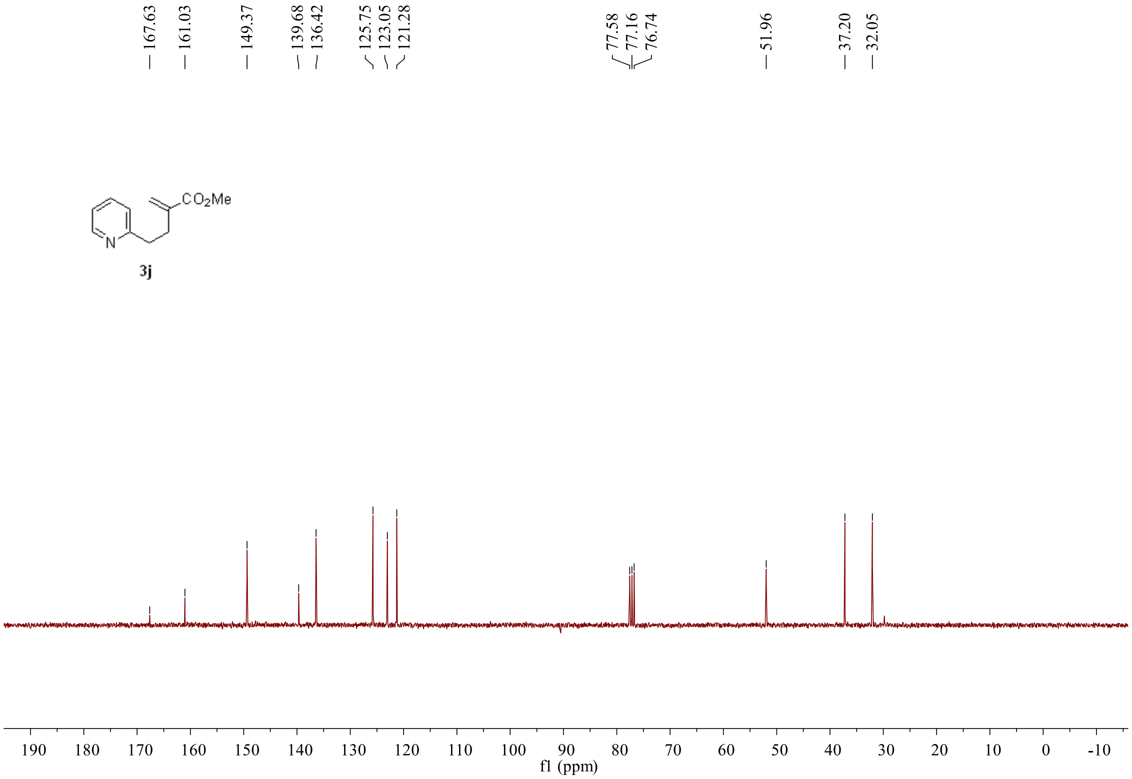

^{1}H NMR spectra of compound 3k

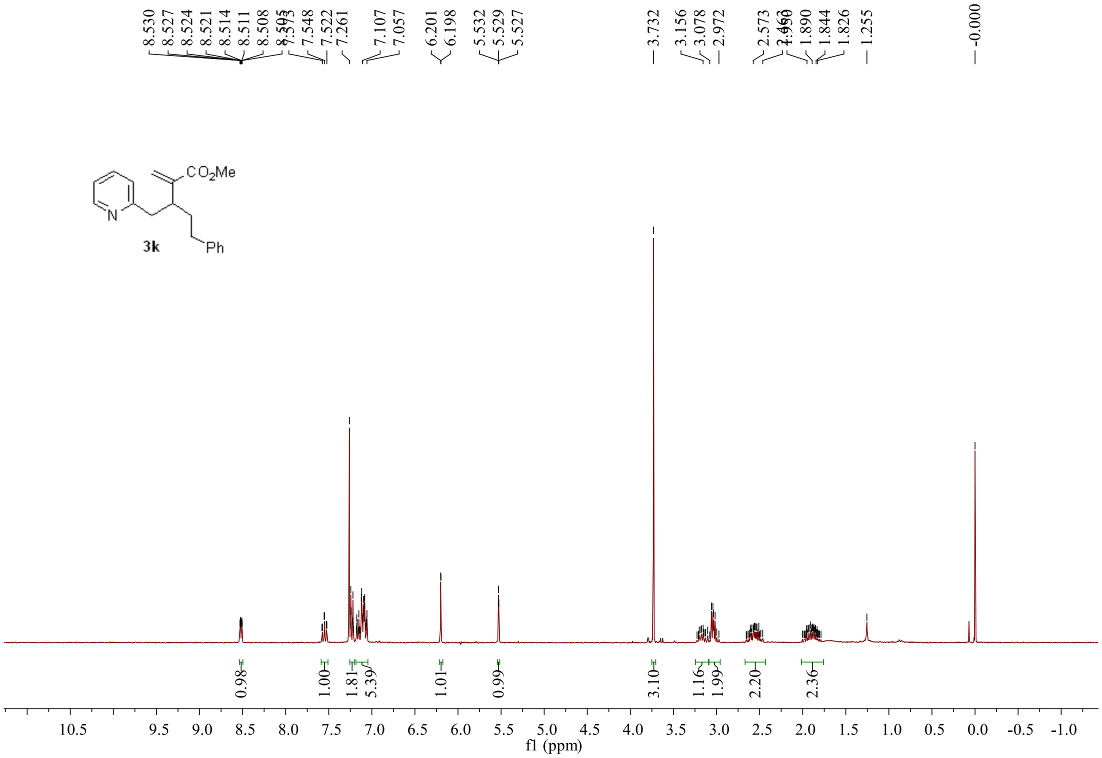

^{13}C NMR spectra of compound 3k

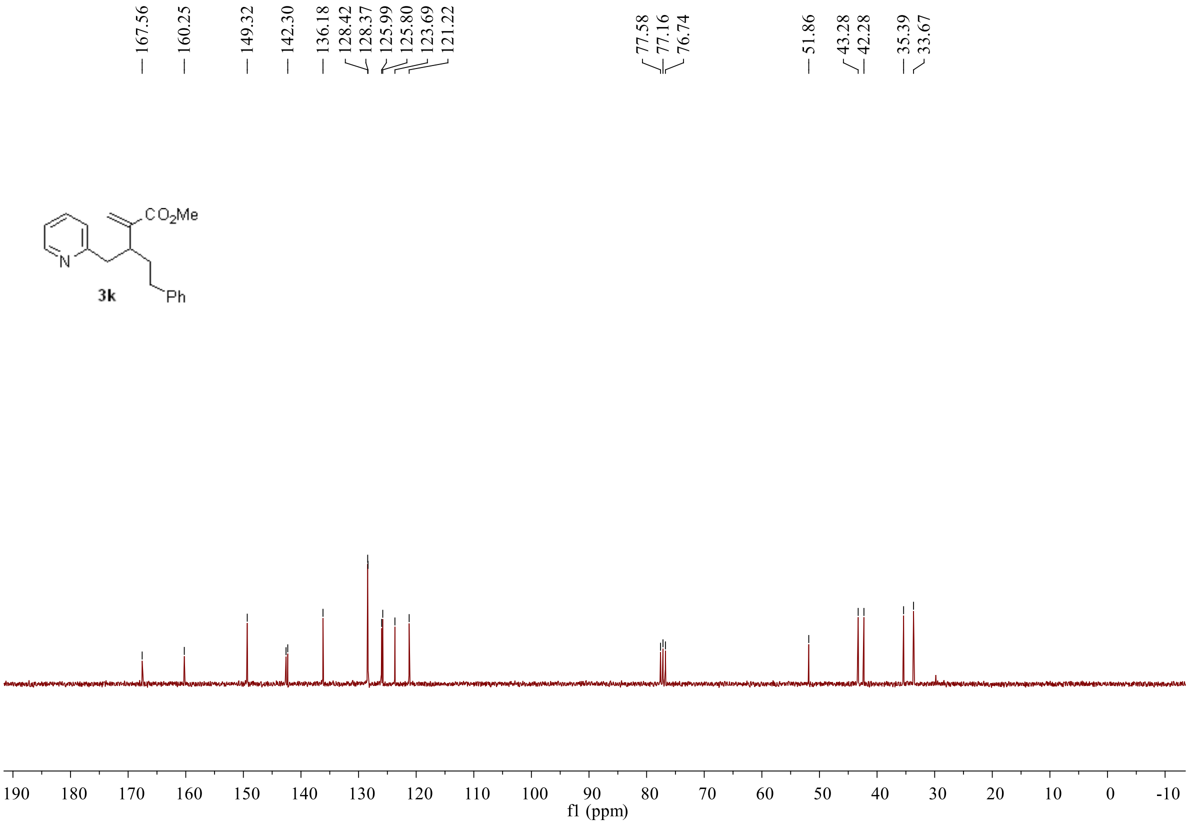

^{1}H NMR spectra of compound 3l

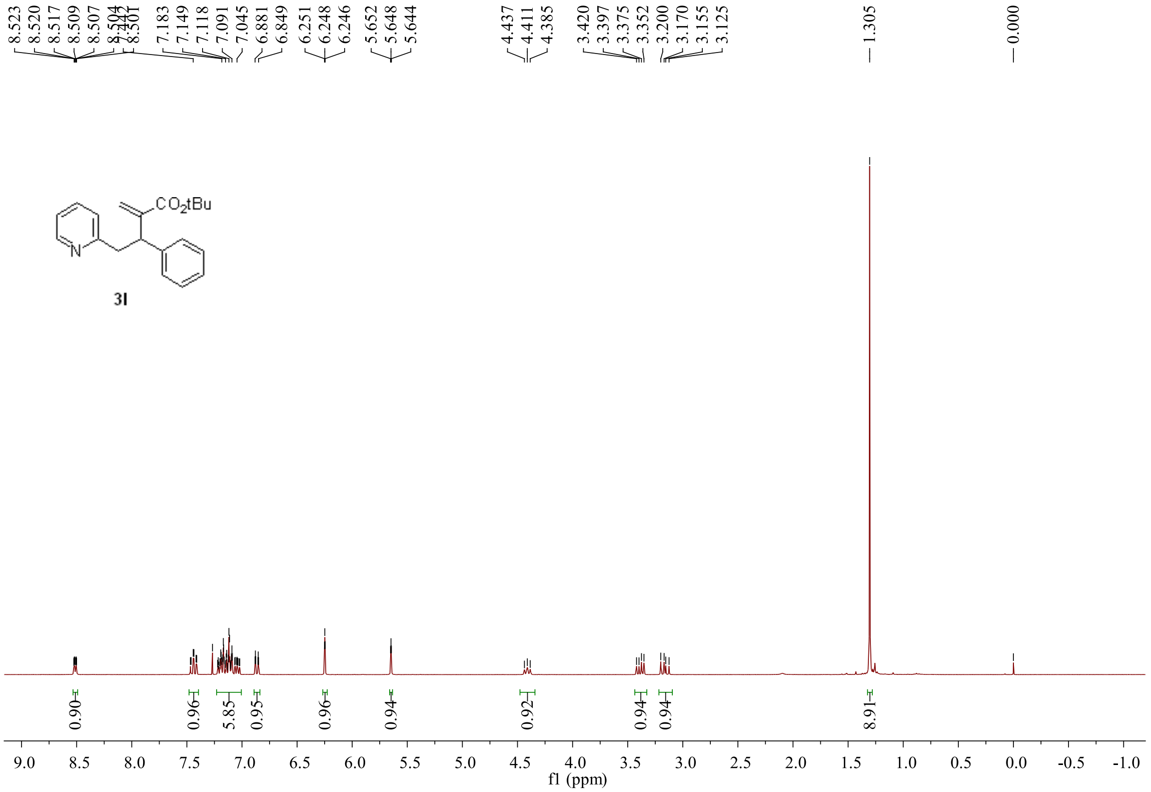

^{13}C NMR spectra of compound 3l

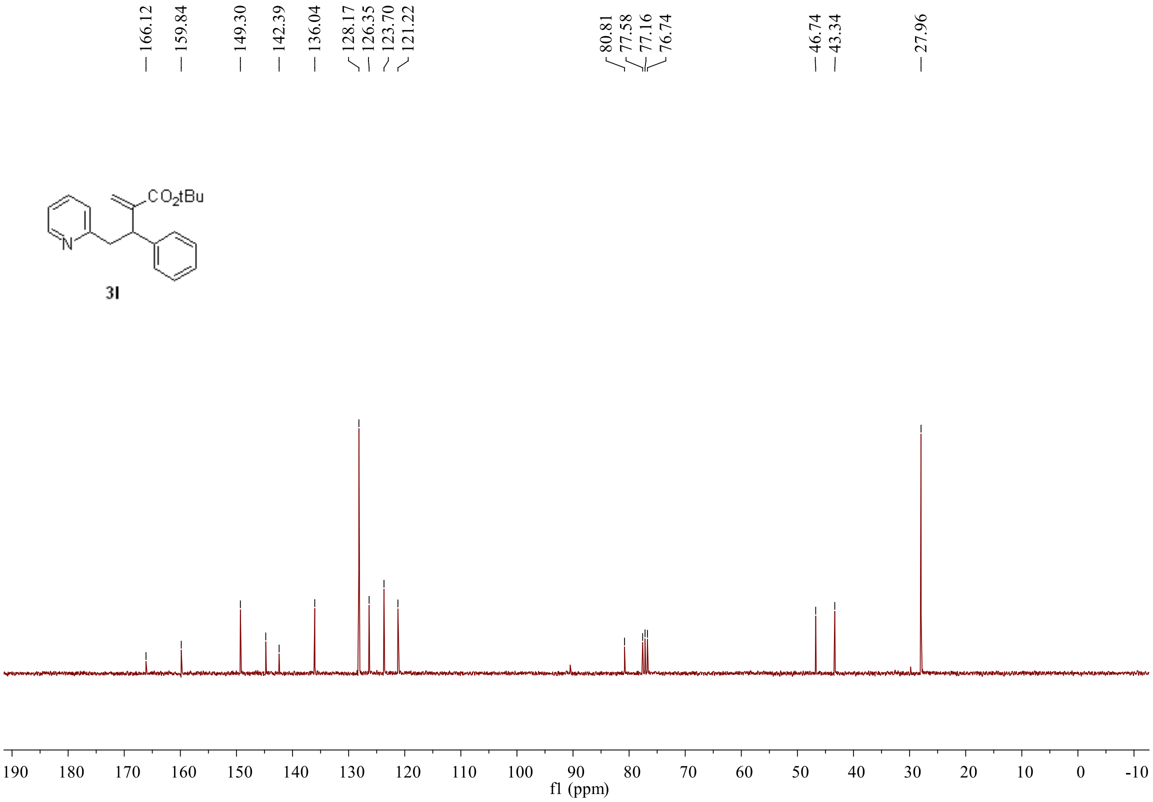

^{1}H NMR spectra of compound 3m

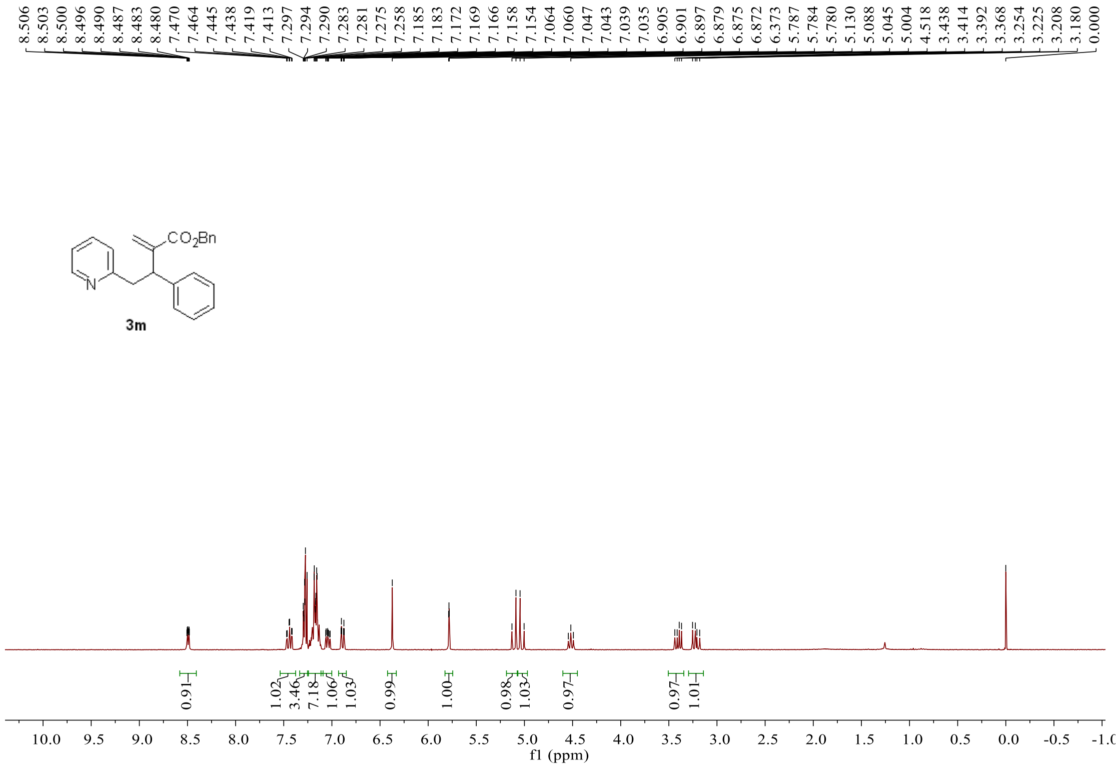

^{13}C NMR spectra of compound 3m

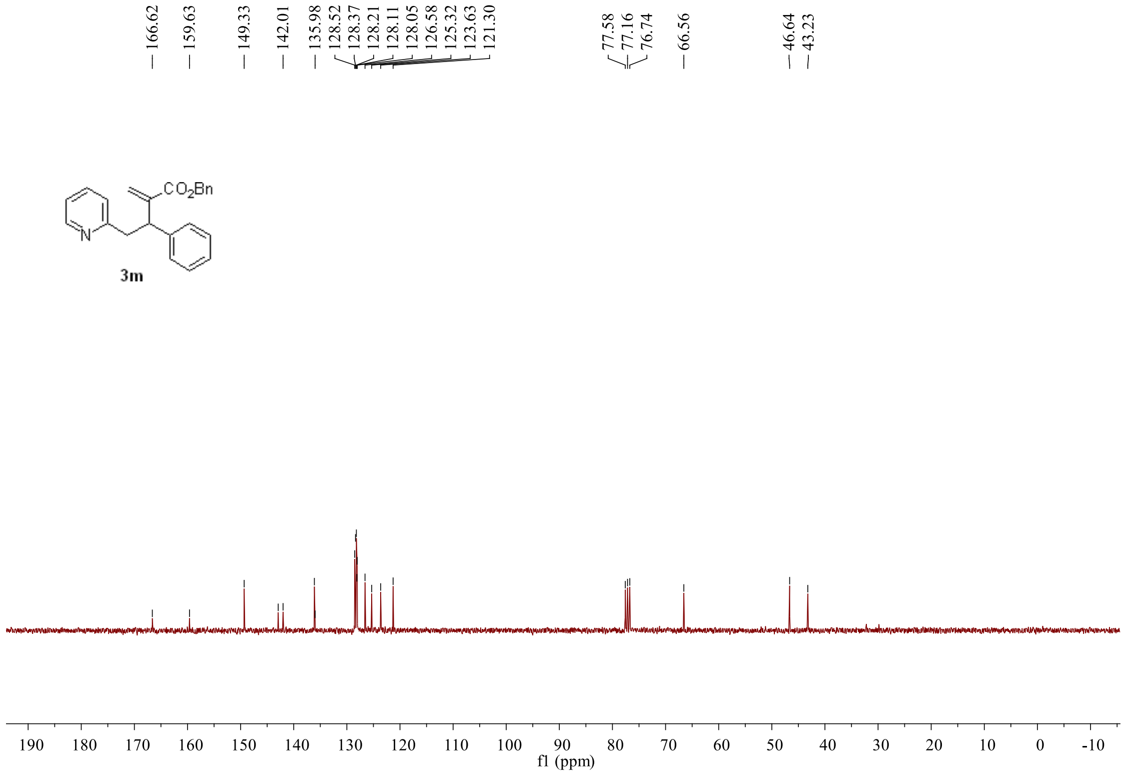

^{1}H NMR spectra of compound 3n

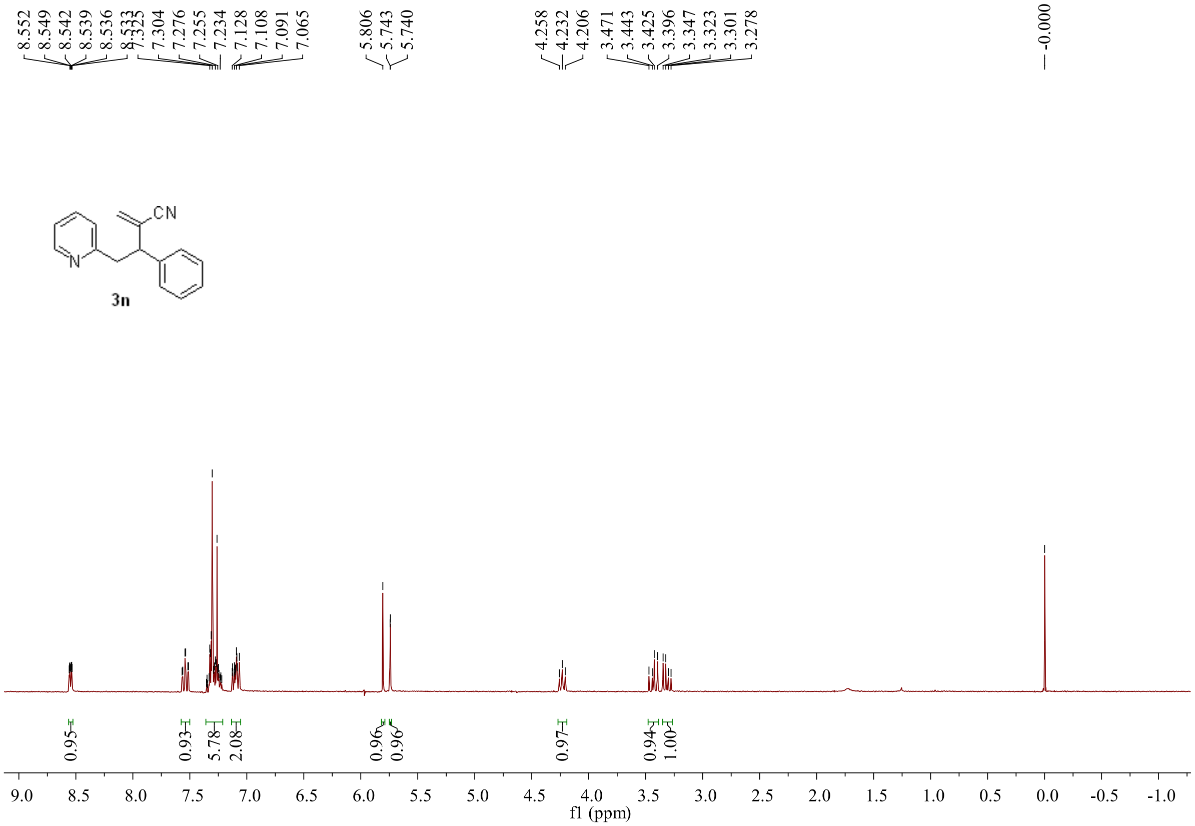

^{1}H NMR spectra of compound 4a

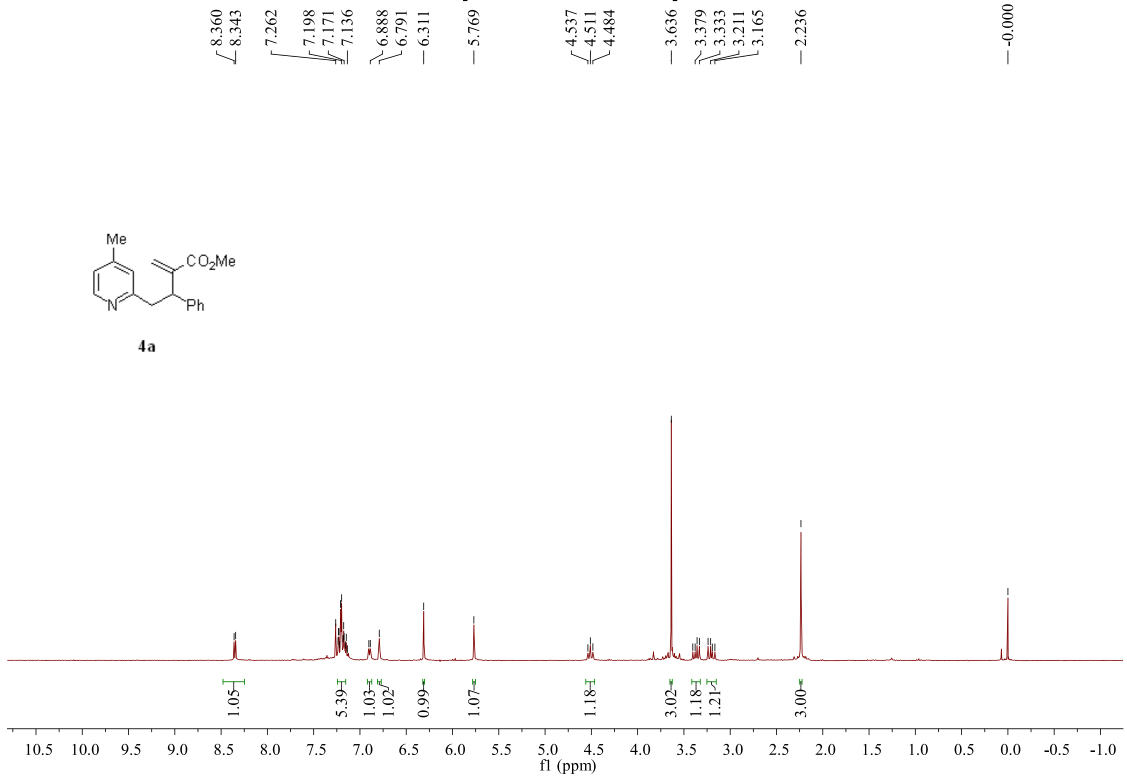

^{13}C NMR spectra of compound 4a

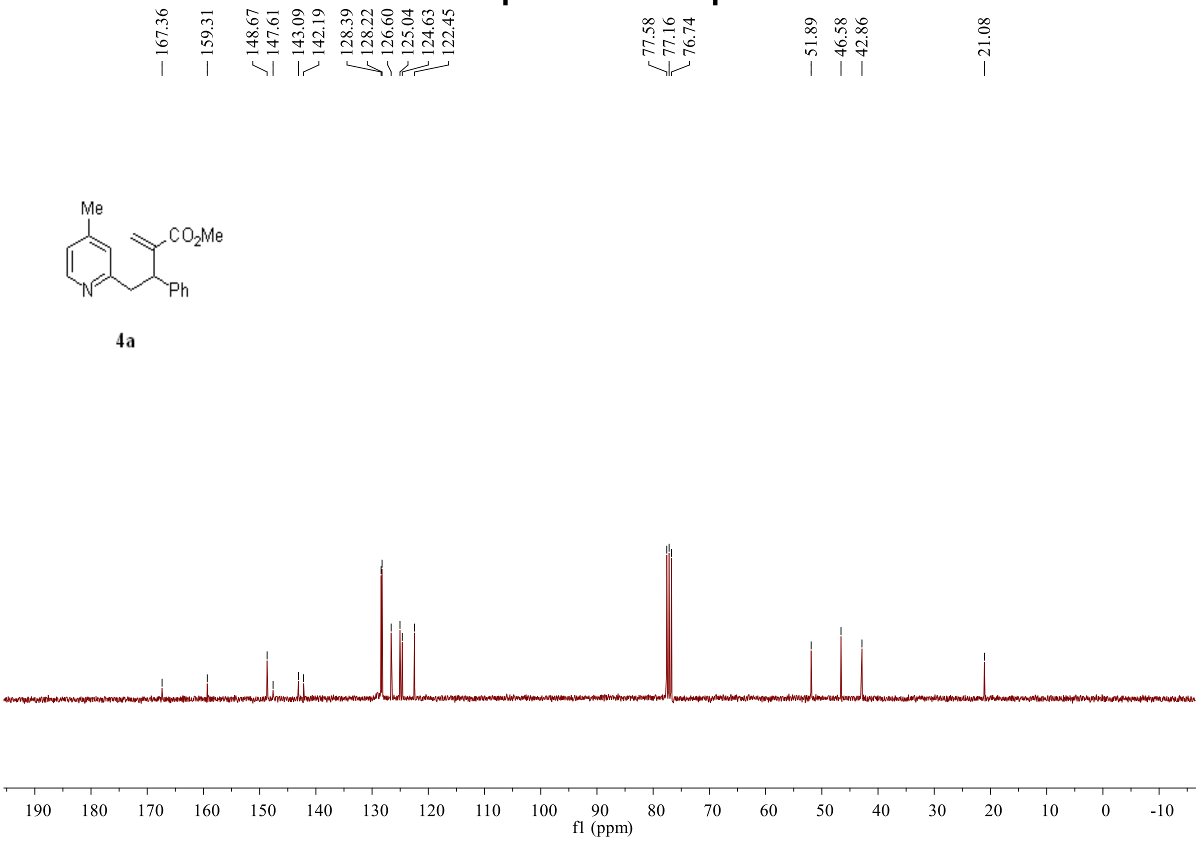

^{1}H NMR spectra of compound 4b

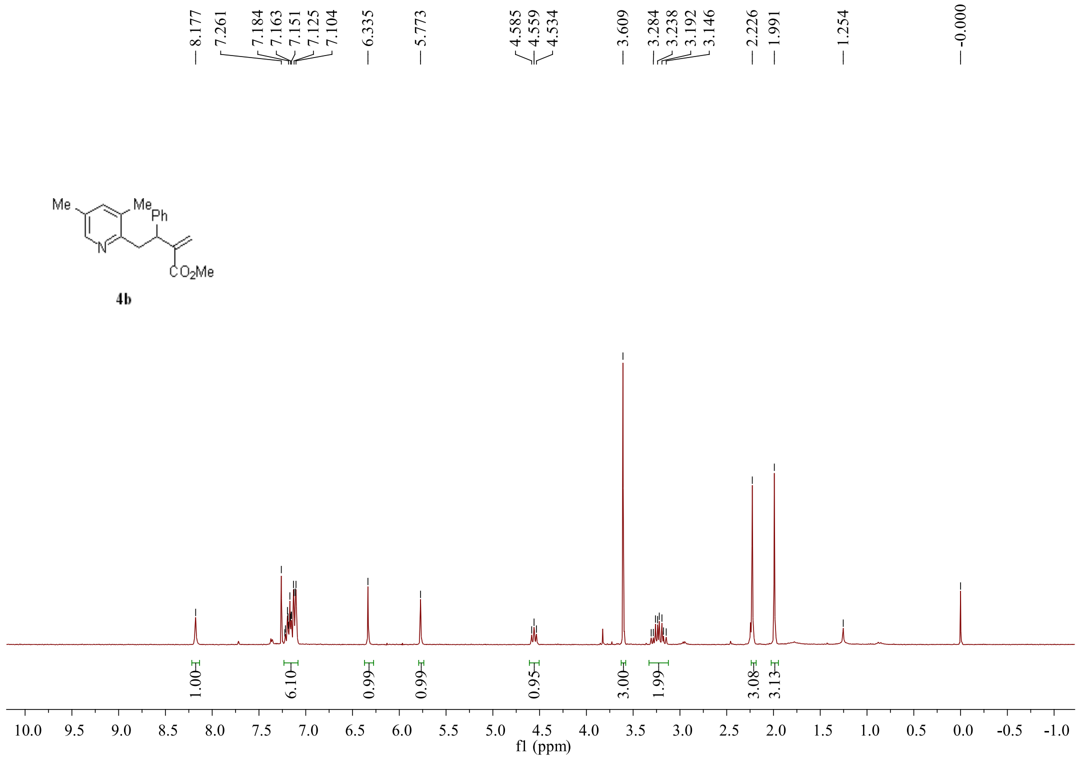

^{13}C NMR spectra of compound 4b

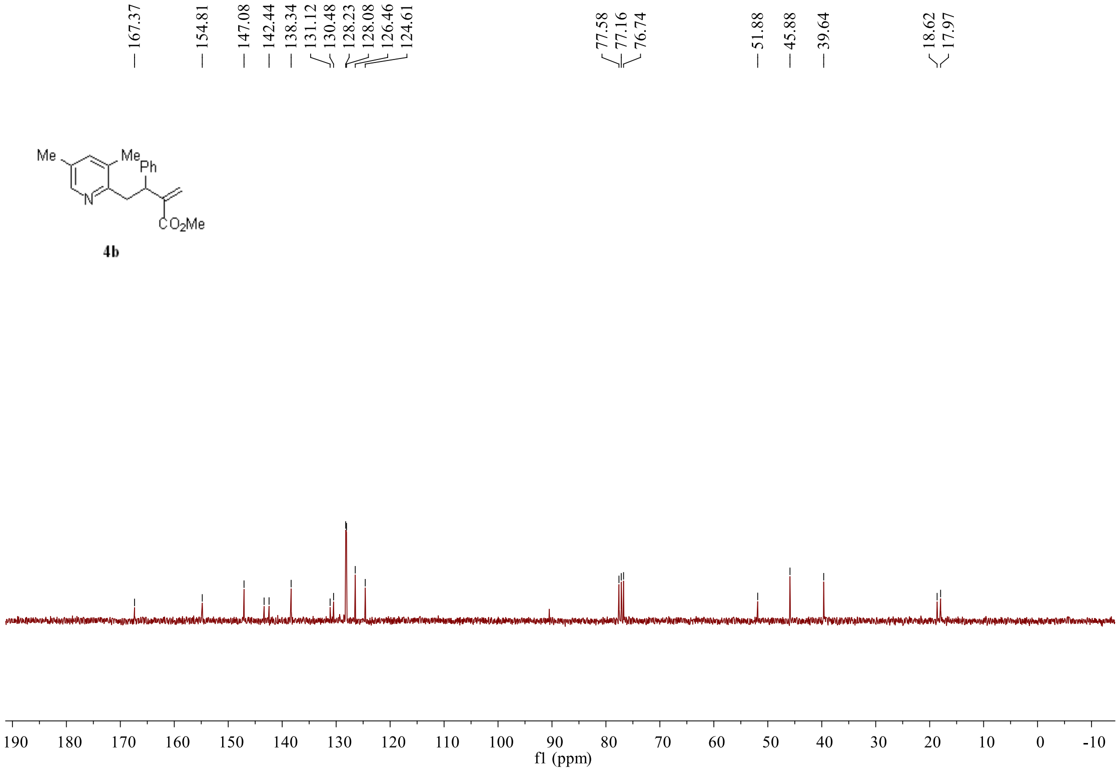

^1H NMR spectra of compound 4c

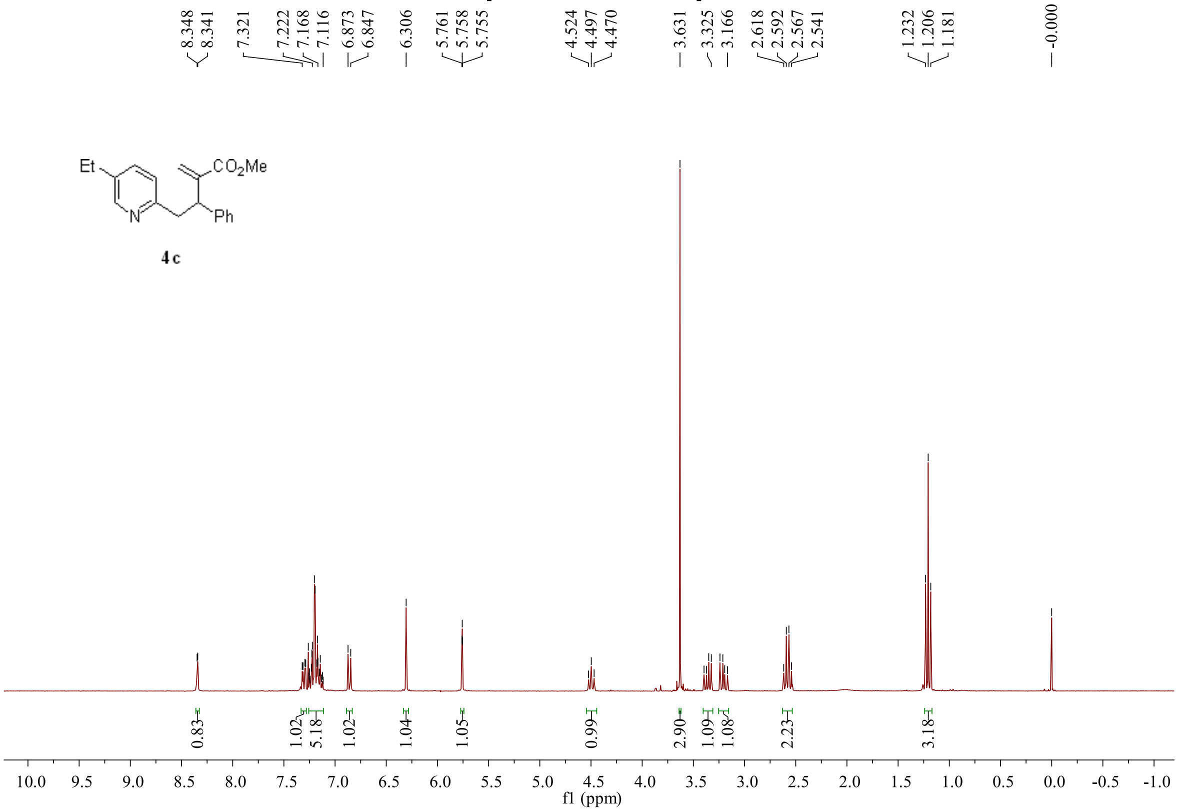

^{13}C NMR spectra of compound 4c

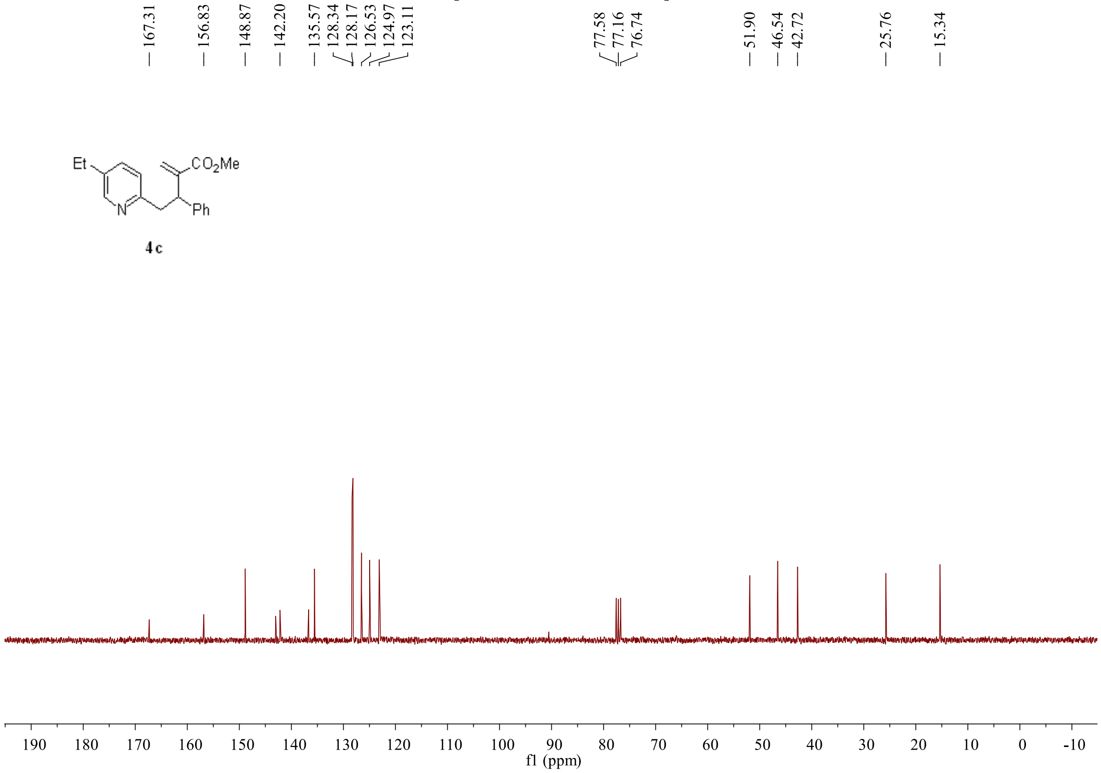

^{1}H NMR spectra of compound 4d

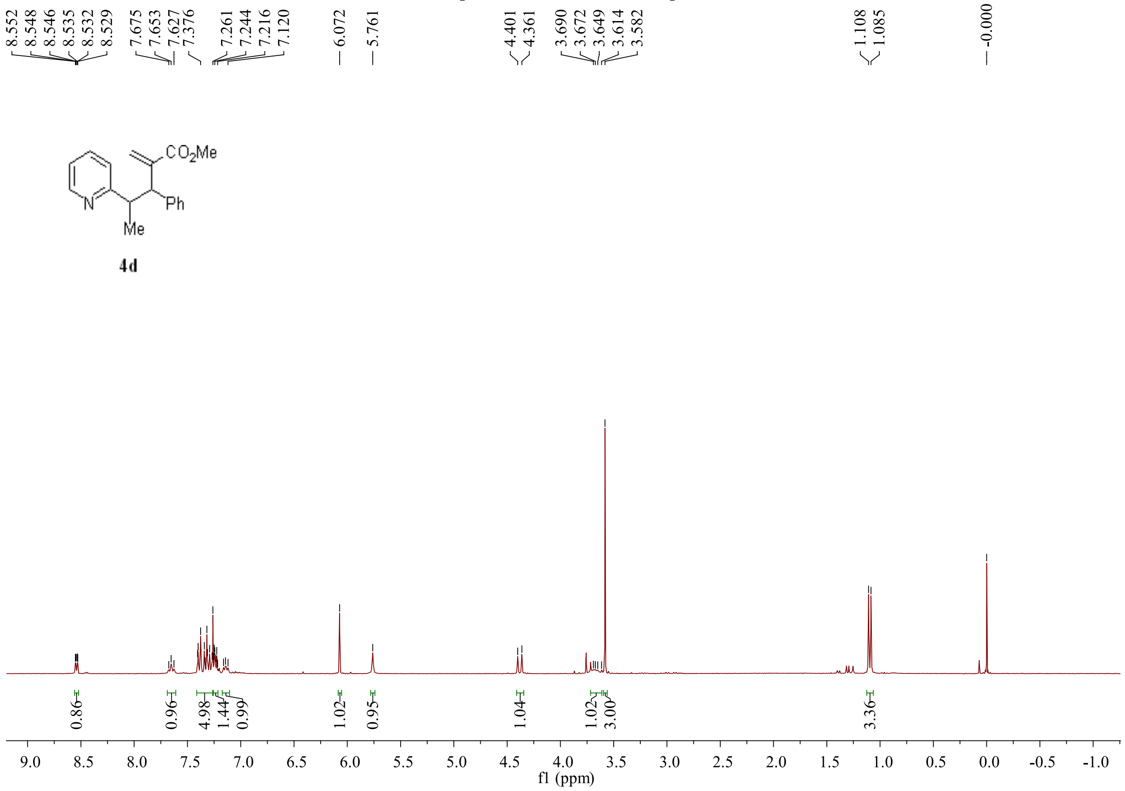

^{13}C NMR spectra of compound 4d

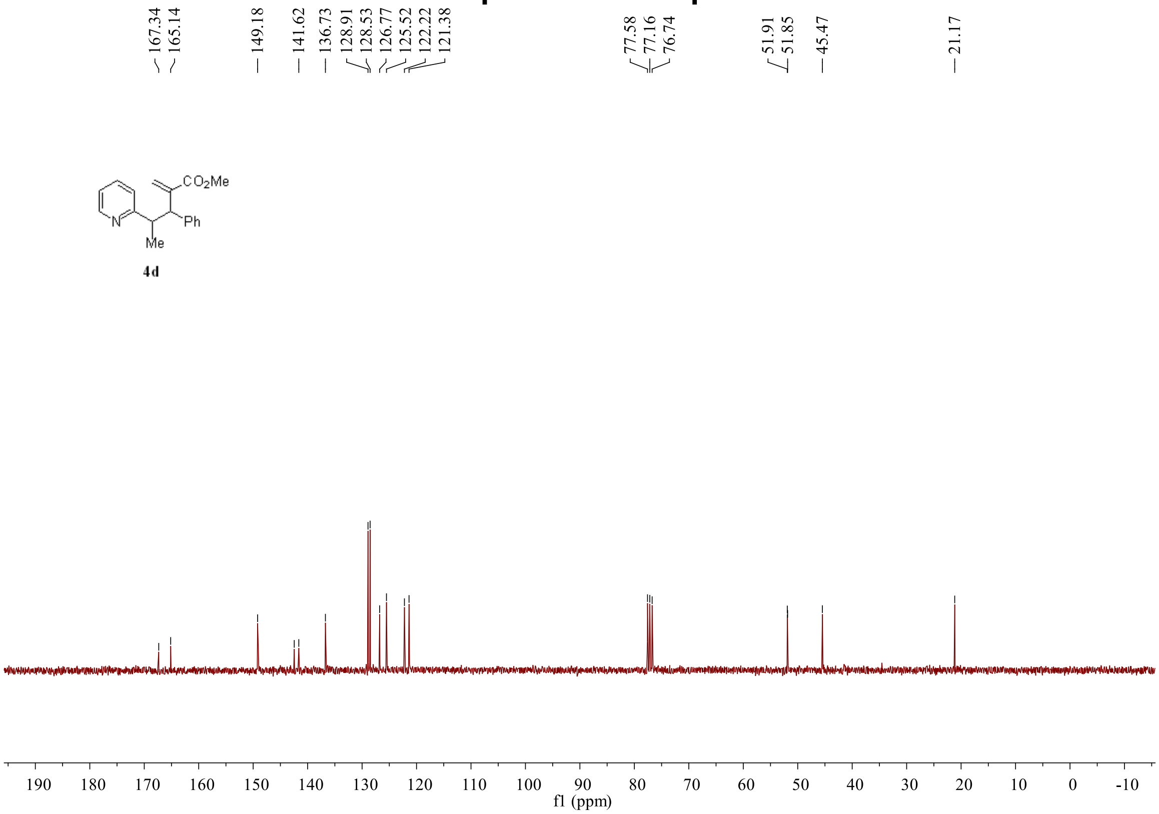

^{1}H NMR spectra of compound 4e

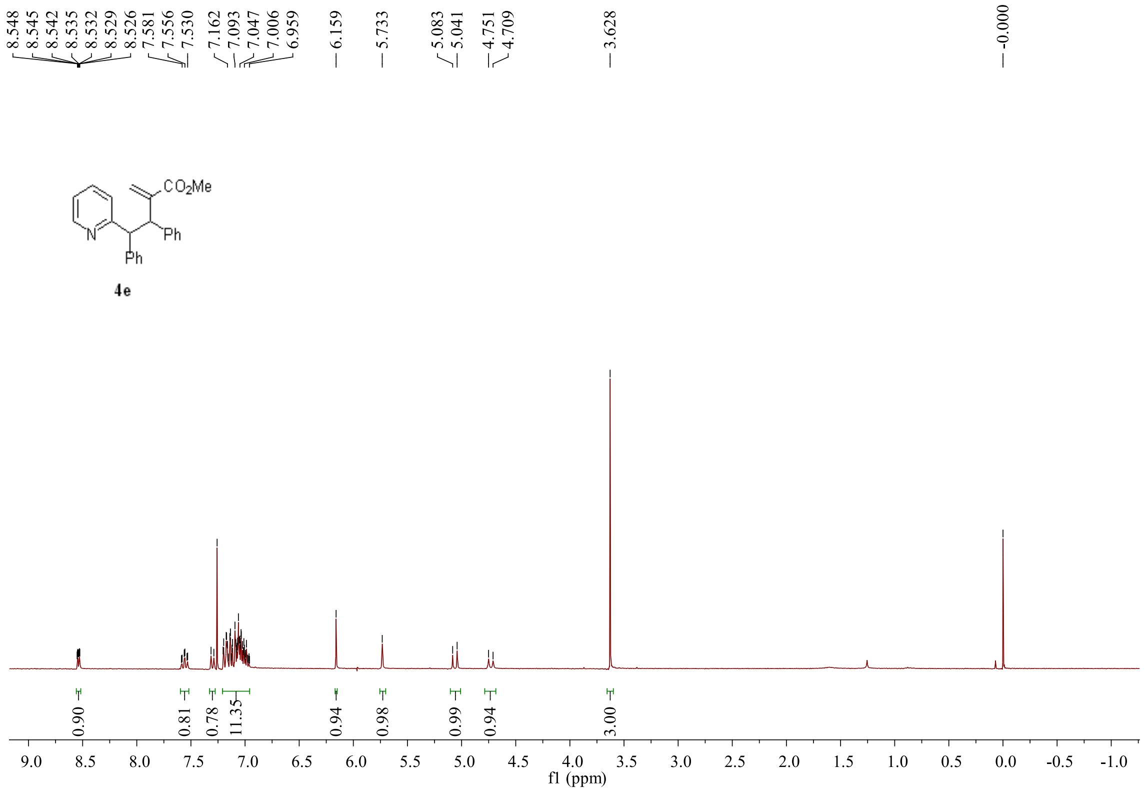

^{1}H NMR spectra of compound 4f

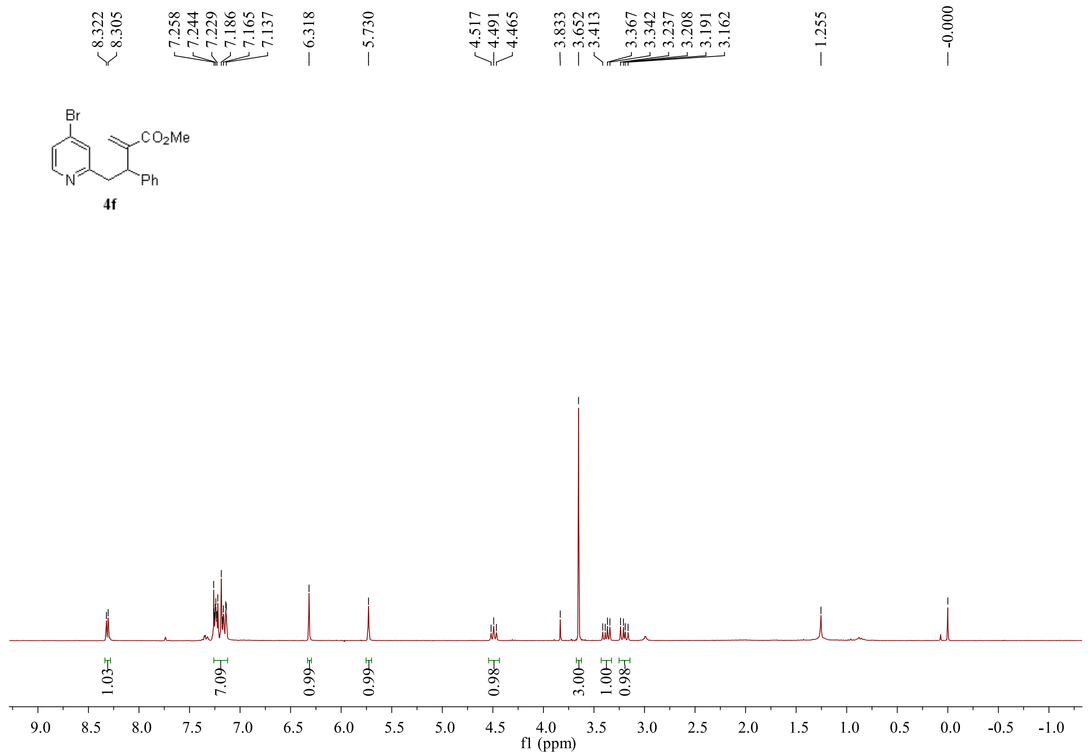

^{13}C NMR spectra of compound 4f

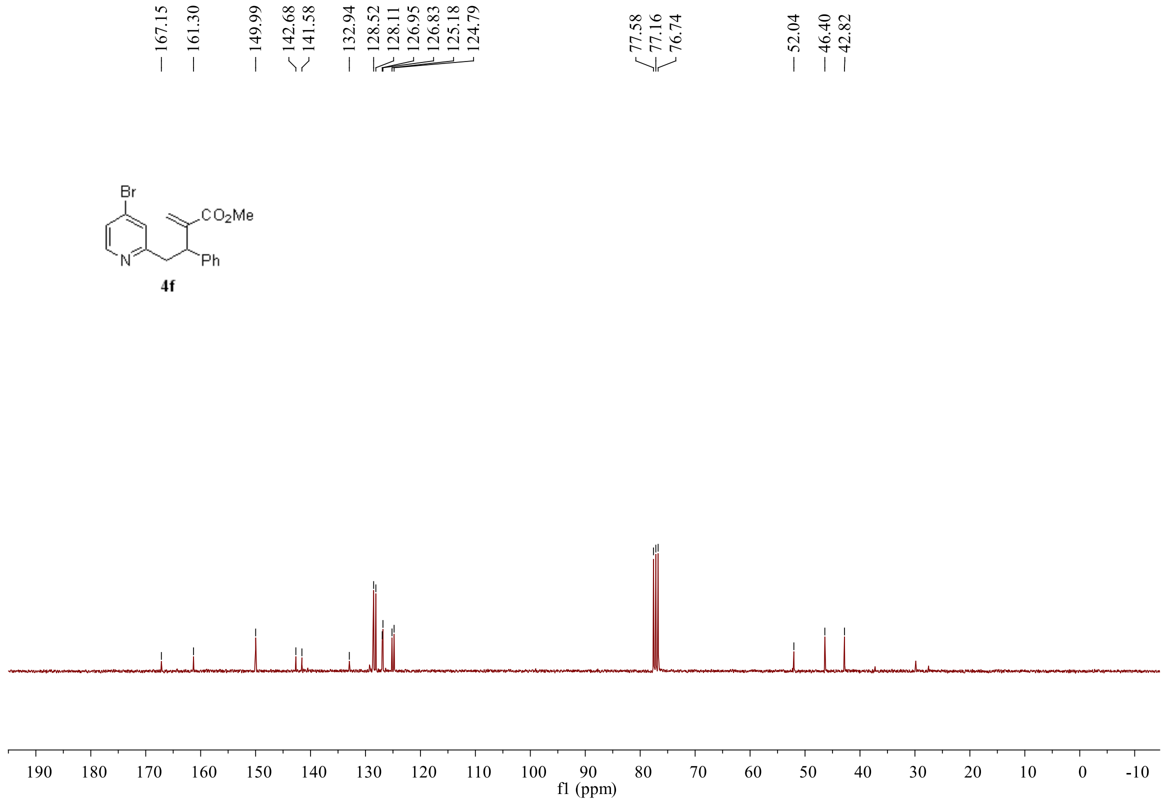

^{1}H NMR spectra of compound 4g

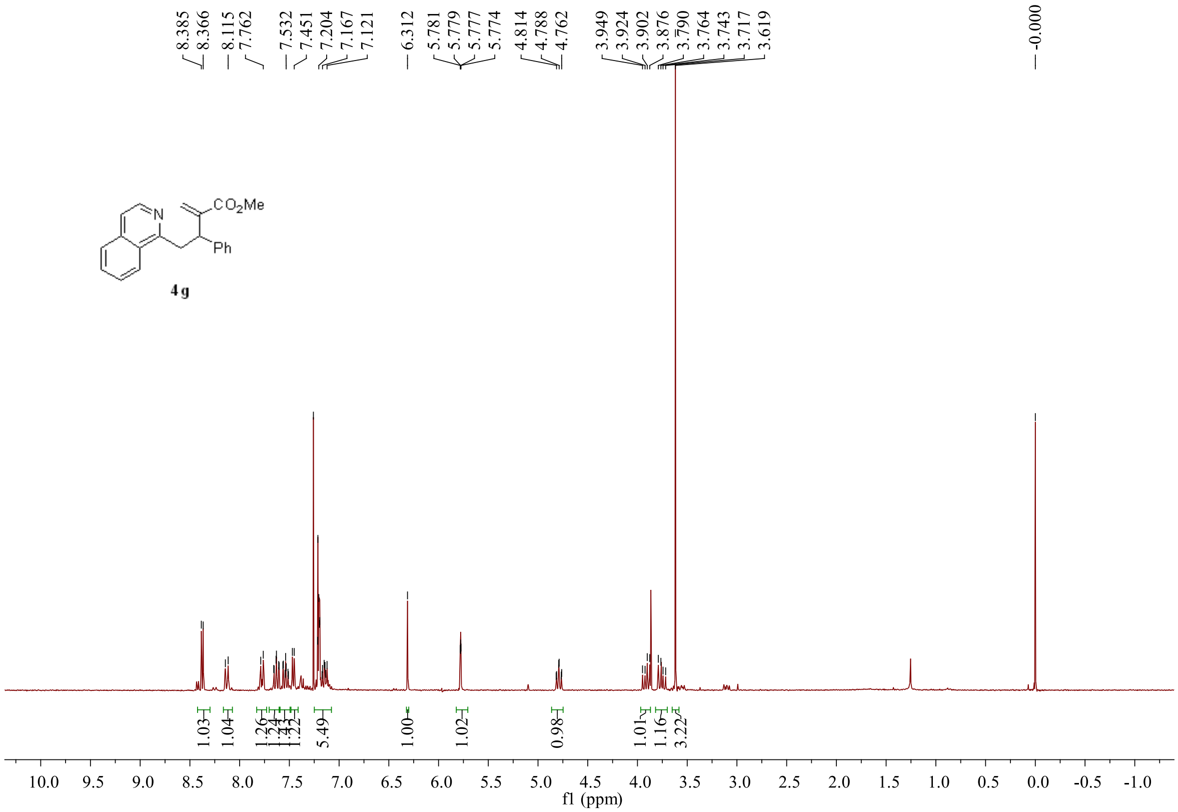

^{13}C NMR spectra of compound 4g

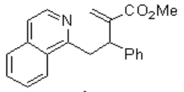

4g

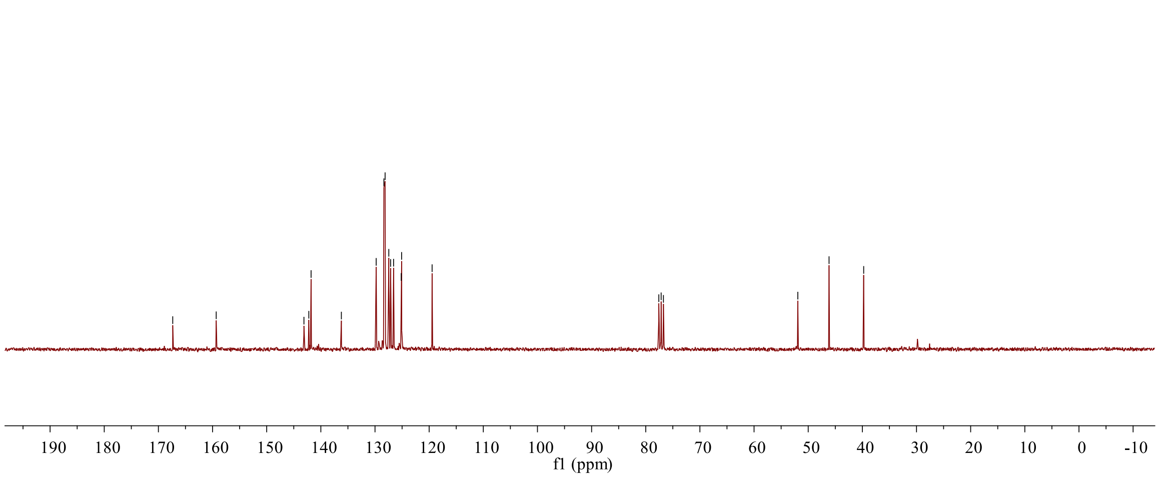

Supplement: File 1 — Experimental details, characterization data and copies of NMR spectra of new compounds. [file Beilstein_J_Org_Chem-17-2505-s001.pdf]
